# Reversible O–H Bond Activation by Tripodal tris(Nitroxide) Aluminum and Gallium Complexes

**DOI:** 10.1021/acs.inorgchem.3c02902

**Published:** 2024-02-22

**Authors:** Joseph S. Scott, Mika L. Maenaga, Audra J. Woodside, Vivian W. Guo, Alex R. Cheriel, Michael R. Gau, Paul R. Rablen, Christopher R. Graves

**Affiliations:** † Department of Chemistry & Biochemistry, Swarthmore College, 500 College Avenue, Swarthmore, Pennsylvania 19081, United States; ‡ Department of Chemistry, University of Pennsylvania, 231 South 34th Street, Philadelphia, Pennsylvania 19104, United States

## Abstract

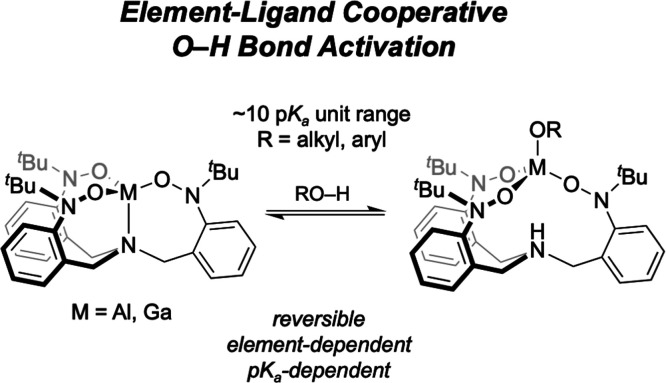

Herein, we report the preparation and characterization of the Group 13 metal complexes of a tripodal tris(nitroxide)-based ligand, designated (TriNOx^3–^)M (M = Al (**1**), Ga (**2**), In (**3**)). Complexes **1** and **2** both activate the O–H bond of a range of alcohols spanning a ∼10 p*K*_a_ unit range via an element-ligand cooperative pathway to afford the zwitterionic complexes (HTriNOx^2–^)M–OR. Structures of these alcohol adduct products are discussed. We demonstrate that the thermodynamic and kinetic aspects of the reactions are both influenced by the identity of the metal, with **1** having higher reaction equilibrium constants and proceeding at a faster rate relative to **2** for any given alcohol. These parameters are also influenced by the p*K*_a_ of the alcohol, with more acidic alcohols reacting both to more completion and faster than their less acidic counterparts. Possible mechanistic pathways for the O–H activation are discussed.

## Abstract 2

Aluminum and gallium complexes supporting a tripodal tris(nitroxide) ligand exhibit reversible, element-ligand cooperative O−H bond activation of various alkyl and aryl alcohols. The equilibrium position of the reactions are affected by both the identity of the group 13 element and the p*K*_a_ of the alcohol, and the aluminum complex reacts both faster and more fully with an alcohol relative to its gallium counterpart

## Introduction

The development of transition-metal coordination complexes designed to undergo the metal–ligand cooperative (MLC) breaking of chemical bonds is an exciting and rich field of research.^1,2^ Of particular importance have been the development of systems for the splitting of polar H–X (X = heteroatom) bonds, which serves as an important step to introducing these reagents into catalytic processes without formal metal-based oxidative addition. This breadth of success is in contrast to the element-ligand cooperative (ELC) chemistry of the main-group elements, which has been much less developed.^3^ We have been investigating the coordination chemistry of aluminum and other group 13 metal complexes supporting redox-active and/or noninnocent ligand frameworks,^4,5^ and specifically, have an interest in understanding the role that the complexes play in ELC chemistry.

**Scheme 1:**
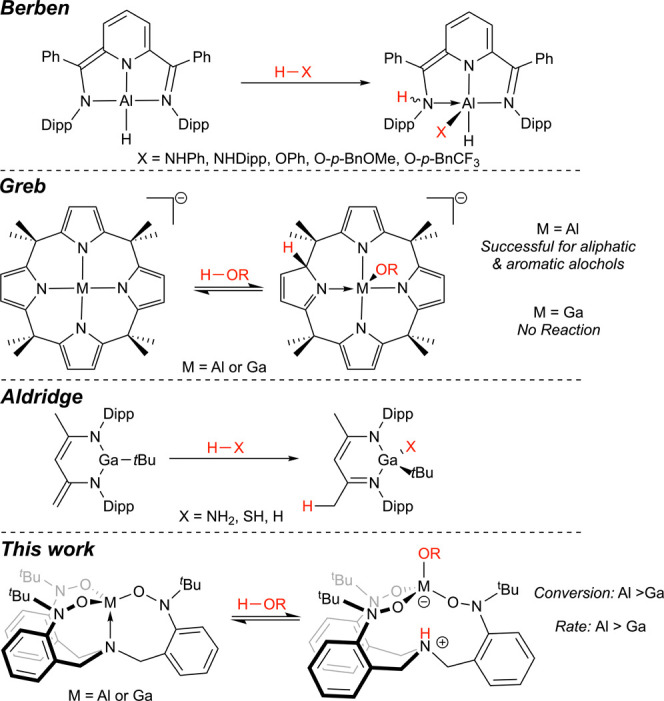
Examples of ELC Reactivity for Aluminum and Gallium Complexes with Polar H–X Bonds, Including This Work (Bottom)

There is precedent for ELC chemistry between aluminum and H–X bonds (Scheme 1). The Berben group has shown that their bis(imino)pyridine aluminum hydride complex (^Ph^I_2_P^2–^)AlH undergoes ELC chemistry with select anilines^6^ and alcohols^7^ to form the (^Ph^HI_2_P^1–^)Al(X)H complexes, where X represents an alkoxo or amido ligand that is installed at the metal ion while the ligand is protonated. They have advanced this chemistry to develop catalytic systems for the dehydrocoupling of amines,^6^ the dehydrogenation of formic acid,^8^ and carbonyl transfer hydrogenation.^9^

Recently, the Greb group reported the preparation of a methylcalix[4]pyrrolato aluminate complex^10^ and described its ELC chemistry,^11^ including its reactivity with alcohols.^12^ The complex reacts with a variety of aliphatic and aromatic alcohols via a reversible ELC process that involves dearomatization/rearomatization of the calix[4]pyrrole ligand to facilitate the protonation/deprotonation step. Although also shown to undergo ELC with carbon dioxide, the corresponding calix[4]pyrrolato gallate complex does not react directly with *i*-PrOH, which the authors attributed to differences in Lewis acidity between the metal ions.^13^

The Aldridge group has demonstrated ELC chemistry for their β-diketiminato galium complex (Dipp_2_Nacnac’)Ga(*^t^*Bu), which reacts with a range of H–X bonds to give the [(Dipp_2_Nacnac)Ga(*^t^*Bu)X] (X = NH_2_, SH, H) complexes.^14^ The reactivity was applicable to H–X bonds of varying polarities and laid the basis for the catalytic reduction of carbon dioxide to MeOBpin by HBpin.

We have reported the synthesis of the (TriNOx^3–^)Al (**1**, TriNOx^3–^ = [{(2-^t^BuNO)C_6_H_4_CH_2_}_3_N]^3–^) complex and showed that it is an effective catalyst for the hydroboration reaction of carbonyl compounds to their boronic esters with HBpin.^15^ Complex **1** combines a Lewis acidic aluminum ion along with several basic sites within the TriNOx^3–^ ligand framework, and we proposed an ELC pathway involving synergistic activation of both the carbonyl (at the Al^3+^ ion) and borane (at a nitrogen atom of a N–O arm of the TriNOx^3–^ ligand) for the hydroboration reaction. In our exploration of synthetic routes to **1**, we discovered that the TriNOxH_3_ ligand precursor reacts incompletely with trimethylaluminum at room temperature to give the complex (HTriNOx^2–^)AlMe, which when heated undergoes a third deprotonation to liberate methane and give **1**. We postulated that reaction of **1** with a polar H–X reagent would result in the analogous (HTriNOx^2–^)AlX complexes via an ELC pathway, where the Lewis acidic aluminum would accept the electrons from the heteroatom X and the TriNOx^3–^ ligand would accept the proton.

Herein, we report the synthesis and characterization of the aluminum and gallium (TriNOx^3–^)M complexes and discuss their reactivity with alcohols to give the (HTriNOx^2–^)M–OR complexes. Unlike with Greb’s gallate complex, our gallium system undergoes ELC directly with alcohols and we are able to compare and contrast reactivity between the two metal ions across a range of protic substrates. We show that the aluminum complex reacts both faster and with greater completion with a given alcohol relative to the same reaction with gallium and develop a mechanistic description supported by a kinetic analysis.

## Results and Discussion

### Synthesis and Characterization of (TriNOx)M Complexes

**Scheme 2:**
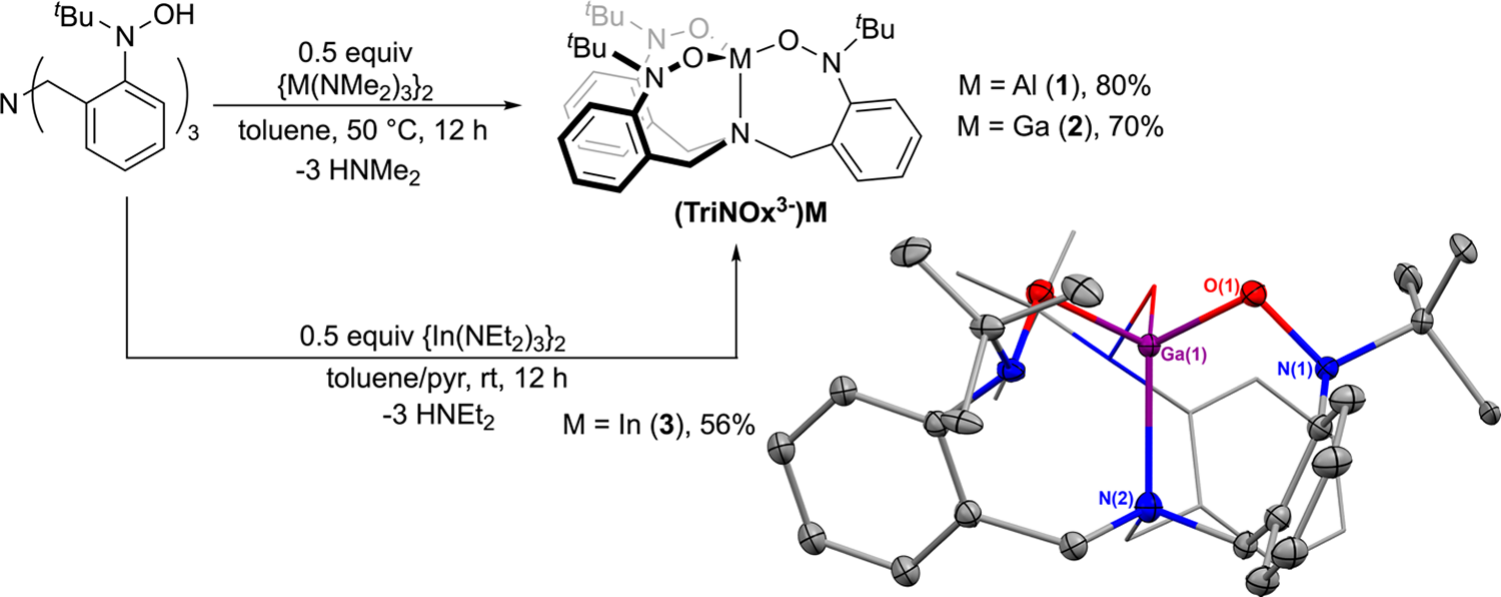
Synthesis of the (TriNOx^3–^)M Complexes **1**–**3** and Solid-State Structure of the (TriNOx^3–^)Ga (**2**) Complex. Ellipsoids are projected at the 50% probability, and H atoms are omitted for clarity. One of the ligand arms is depicted using a wireframe model. *R*_1_ = 0.0637.

The (TriNOx^3–^)M (M = Al (**1**); Ga (**2**)) complexes are most easily prepared from the reaction between {M(NMe_2_)_3_}_2_ with two equivalents of TriNOxH_3_ ligand precursor in toluene (Scheme 2). After heating the reaction mixtures for 12 h at 50 °C, complexes **1** and **2** can be cleanly isolated from the reactions following the removal of volatiles, giving off-white solids in average yields of 80 and 70%, respectively.^16^ This synthetic route is an improvement over our previously reported preparation of **1-py** (py = pyridine) in which salt metathesis was used to install the TriNOx^3–^ ligand.^15^ The salt metathesis method was unsuccessful in the preparation of **2** as the isolation of pure (TriNOx^3–^)Ga from the reaction byproducts was always complicated by partial decomposition of the complex into some unknown material. **1** and **2** are both indefinitely stable in the solid state when stored under a nitrogen atmosphere at −80 °C, although they do decompose if stored in a glovebox over the course of weeks to months if care is not taken to protect the complexes from volatile reagents, regardless of the temperature at which they are stored.^17^**1** and **2** are soluble in hydrocarbon solvents such as toluene and benzene as well as in more polar solvents such as tetrahydrofuran, chloroform, and methylene chloride but have minimal solubility in acetonitrile, pentane, or hexanes.

We also prepared the (TriNOx^3–^)In (**3**) complex through the reaction of {In(NEt_2_)_3_}_2_ with two equivalents of TriNOxH_3_ in a toluene/pyridine mixture at room temperature (Scheme 2). Following evaporation of the solvents, complex **3** was collected in 56% yield after purification by precipitation from a concentrated pyridine solution at −25 °C. **3** is much less soluble than its aluminum and gallium counterparts; it has very limited solubility even in polar solvents like THF or dichloromethane and gives homogeneous solutions only in boiling pyridine. Based on this limited solubility, complex **3** was not included in the alcohol reactivity studies carried out with the other (TriNOx)M complexes (vide infra).

Complexes **1**–**3** were readily characterized by ^1^H and ^13^C NMR spectroscopies. All of the complexes exhibit a single resonance in their ^1^H NMR spectrum assignable to the ^*t*^Bu groups of the ligand as well as four sets of aromatic resonances that each integrate to three protons, indicating 3-fold symmetry of the tripodal ligand when bound to the group 13 metal ions. In all cases, the protons of the bridgehead CH_2_ groups in **1**–**3** are diastereotopic and are assignable as two doublets (*J* = 11–12 Hz) in the ^1^H NMR spectrum, each integrating to three protons. The ^13^C{^1^H} NMR spectra for the complexes each have six unique aromatic resonances along with signatures for both the ^*t*^Bu substituents and methylene bridgehead carbons of the TriNOx^3–^ ligand.

Single crystals of the (TriNOx^3–^)Ga complex were grown from a concentrated THF solution layered with hexanes at −25 °C, allowing for the characterization of **2** by X-ray crystallography. The molecule lies on a 3̅ rotary inversion axis that passes through the gallium ion and basal nitrogen atom (N(2)), and there are multiple types of disorder resulting in a total of four superimposed molecules in the asymmetric unit. A representation of one of these molecules is shown in Scheme 2. Full details of the various disorders and their modeling are available in the Supporting Information. The gallium ion in **2** sits within the ligand core and is coordinated by all three oxygen atoms of the nitroxide groups and the bridgehead nitrogen in a tetrahedral geometry (τ_4_ = 0.94). The Ga–O (1.854(9) Å) distances in **2** are comparable to the Ga–O distances in the others structurally characterized, 4-coordinate gallium ions supporting a NOOO primary coordination sphere,^18^ although our Ga–N (2.22(2) Å) is somewhat longer than the Ga–N distances for the same comparison group.

We were surprised by the absence of coordinated Lewis base at the gallium ion in the solid-state structure of **2** given that **1** crystallizes as its base adduct with pyridine.^15^ With this in mind, we used the Gutmann–Beckett method^19,20^ to evaluate the Lewis acidity of **1** and **2** in solution. Attempts to collect similar data for complex **3** were inhibited by lack of solubility of **3** in either CDCl_3_ or CD_2_Cl_2_. The difference in ^31^P chemical shift (Δδ) of Et_3_PO·(TriNOx)Al and Et_3_PO measured in C_6_D_6_ is 19.7 ppm. In contrast, we do not observe a Δδ between free Et_3_PO and the **2**/Et_3_PO mixture, suggesting that the gallium ion does not coordinate the Lewis base in solution. We carried out the analogous experiments using Et_3_PS in place of Et_3_PO. In this case, we do not observe a Δδ between the Et_3_PS and its mixture with either **1** or **2**, suggesting that the lack of Lewis acidity of **2** is not solely due to a mismatch in the hardness of Et_3_PO. At this point, we are unsure where the difference in Lewis acidity originates, but we do suggest that this difference plays a fundamental role in the reactivity of **1** and **2** with alcohols, concepts which we explore in more detail throughout the remainder of this paper.

### Reactivity of the (TriNOx^3–^)M Complexes with ROH

**Scheme 3:**
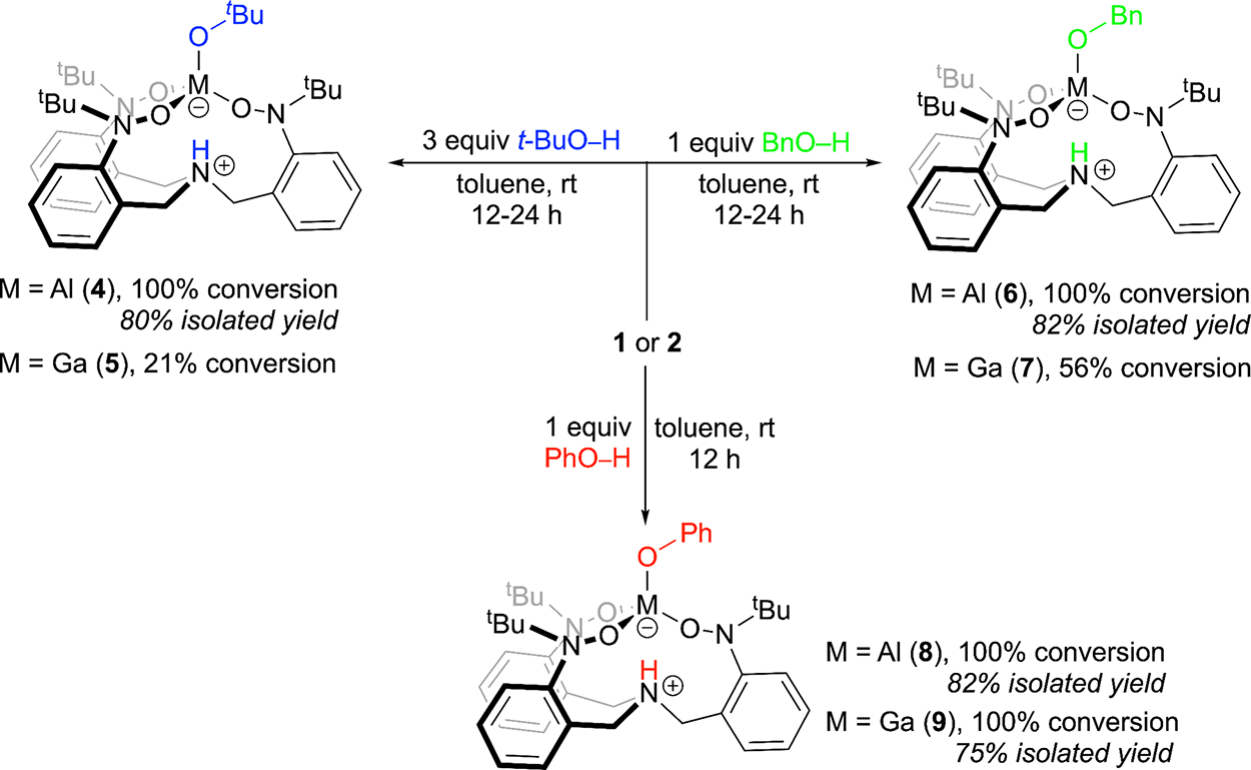
Reactivity of the (TriNOx^3–^)M Complexes **1** (M = Al) and **2** (M = Ga) with Alcohols to Give the Complexes (HTriNOx^2–^)M–OR **4**–**9**

We investigated the reactivity of the (TriNOx^3–^)M complexes **1** and **2** with various alcohols (Scheme 3). (TriNOx^3–^)Al was reacted with *tert*-butanol (*t-*BuOH) in toluene at room temperature to give the Zwitterion (HTriNOx^2–^)Al–O^*t*^Bu (**4**) in which the O–H bond of the alcohol has reacted to install a *tert*-butoxy ligand at the metal ion and protonate the bridgehead nitrogen of the TriNOx^3–^ ligand. Using one equivalent of *t-*BuOH gives **4** in 86% conversion after 24 h,^21^ but the reaction can be pushed to completion by increasing the amount of alcohol to three equivalents. Using these conditions, **4** was isolated in 80% yield after 12 h following removal of volatiles. In contrast, the reaction between (TriNOx^3–^)Ga (**2**) and *t-*BuOH is much less successful. Reaction of **2** with three equivalents of *t-*BuOH in toluene gives (HTriNOx^2–^)Ga–O^*t*^Bu (**5**) in only 21% conversion after 24 h. The conversion improves only slightly with increasing the equivalents of *t-*BuOH (25% conversion with 6 equiv) and stirring **2** in neat *t*-BuOH results in decomposition of the complex into free TriNOxH_3_ ligand precursor as evidenced by ^1^H NMR spectrocopy.^22^

We next carried out the reaction of the (TriNOx^3–^)M complexes with phenol (PhOH). The 1:1 reaction between **1** or **2** and phenol in toluene at room temperature results in the formation of the alcohol adduct complexes (HTriNOx^2–^)Al–OPh (**8**) and (HTriNOx^2–^)Ga–OPh (**9**), respectively. These reactions occur much faster than the reactions between **1** and **2** with *t-*BuOH, as evidenced by the generation of opaque reaction solitons within minutes in the former. The products **8** and **9** were isolated in 82% and 75% yield, respectively, after the removal of volatiles from the crude reactions after 12 h.

Finally, we investigated the reactivity of **1** and **2** with benzyl alcohol (BnOH). The reaction between **1** with a stoichiometric amount of BnOH in toluene gives (HTriNOx^2–^)Al–OBn (**6**) in 100% conversion after 12 h at room temperature. Following workup, **6** was isolated in 82% yield. Conversely, the reaction of **2** with BnOH gives (HTriNOx^2–^)Ga–OBn (**7**) in only 56% conversion under identical conditions, which is increased only slightly (to 61%) when three equivalents of benzyl alcohol are used. Increasing the amount of alcohol further results in the consumption of the starting material, but free TriNOxH_3_ is also produced along with **7**. Collectively, these results suggest that the identity of the metal ion and the specific alcohol both influence the thermodynamic and kinetic parameters of the reaction between (TriNOx^3–^)M and ROH. In particular, reactions with **1** proceed both faster and in higher conversion relative to the reaction of **2** with the same alcohol. Additionally, for a given metal, as the alcohol becomes more acidic the reaction proceeds both faster and in higher conversion. We more fully examine these dependencies below.

Complexes **4**, **6**, **8**, and **9** are stable for the period of weeks if stored in the solid state under a nitrogen atmosphere at −25 °C, but are not stable in the long term under these conditions. All of the complexes are partially soluble in toluene and soluble in both chloroform and methylene chloride. Complexes **4** and **6** are both also soluble in benzene. Neither **8** or **9** are readily soluble in THF and require several hours of stirring to achieve a homogeneous solution. However, the complexes are not stable in THF and solubilization is always accompanied by partial decomposition of the complexes as judged by the presence of free TriNOxH_3_ in the ^1^H NMR spectra of their solutions. We suspect that none of the complexes **4**, **6**, **8**, and **9** have long-term stability in THF solution, although we have not rigorously tested this hypothesis. The complexes are not stable pyridine, resulting in partial decomposition of the complexes into mixtures of (TriNOx^3–^)M and what we suspect is [(TriNOx^3–^)M–OR][py–H].

Complexes **4**, **6**, **8**, and **9** were characterized by ^1^H and ^13^C NMR spectroscopies. In all cases, the ^1^H NMR spectra of the complexes exhibit a single resonance assignable to the protons of the *^t^*Bu groups of the tripodal ligand. These signals come at chemical shifts ∼0.5 ppm upfield relative to the resonance for the ligand *^t^*Bu groups in the ^1^H NMR spectra of **1** and **2** and suggest *pseudo*-*C*_*3*_ symmetry of the (HTriNOx^2–^)M–OR complexes in solution. The spectra also all display a broad singlet at ∼11 ppm that can be assigned to the N–H proton of the complexes. The diastereotopic protons of the CH_2_ groups in the (HTriNOx^2–^)M–OR complexes appear as a doublet (*J* = 12 Hz) and a doublet-of-doublets (*J* ∼ 12 Hz; 8–12 Hz), the latter splitting pattern of which arises from coupling between the methylene proton with its diastereotopic partner, and the newly formed N–H proton on the bridgehead nitrogen atom. The ^13^C NMR spectra for the complexes each have six unique aromatic resonances assignable to the HTriNOx^2–^ ligand along with signatures for both the ligand *^t^*Bu substituents and methylene carbons. The NMR signatures of the various apical groups of the (HTriNOx^2–^)M–OR complexes are also observed in the ^1^H and ^13^C NMR spectra complexes **4**, **6**, **8**, and **9**. For example, the ^1^H NMR spectra of **4** has a signal at δ 1.81 ppm assignable to the *^t^*Bu group the *tert*-butoxy ligand, and the CH_2_ of the benzyloxy ligand appears as a set of diastereotopic protons in the δ 5.5–5.6 ppm range of in the ^1^H NMR spectrum of **6**.

**Figure 1:**
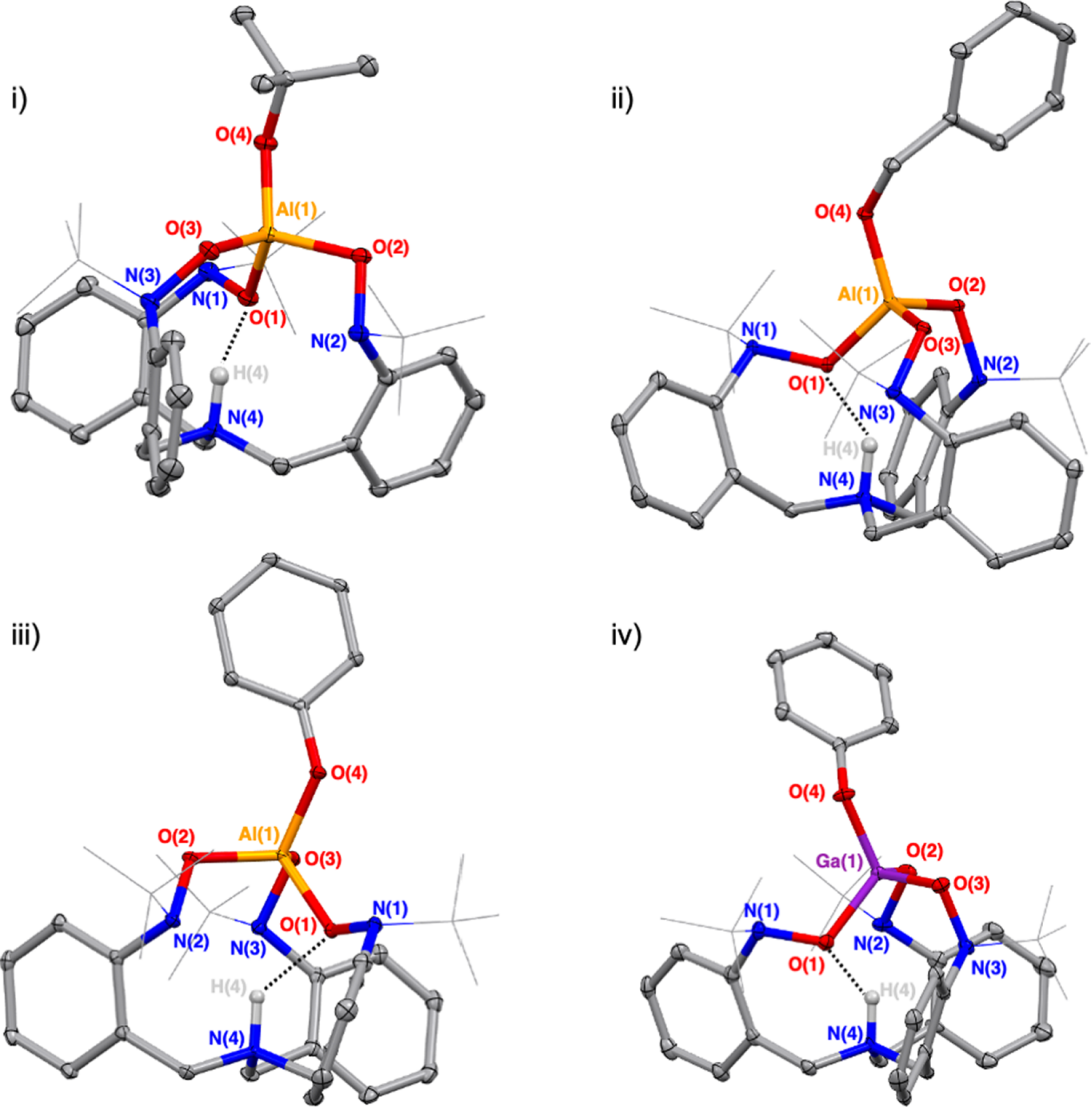
Solid state structures of the (HTriNOx^2–^)M–OR compounds **4**, **6**, **8**, and **9**. Ellipsoids are projected at 30% probability and the *tert*-butyl groups of the TriNOx ligand are shown in wireframe for clarity. With the exception of the N–H, hydrogen atoms have been omitted for clarity. (i) (HTriNOx^2–^)Al–O^*t*^Bu (**4**), *R*_1_ = 0.0427; τ_4_[Al(1)] = 0.95; O(1)---H(4), 1.842 Å. (ii) (HTriNOx^2–^)Al–OBn (**6**), *R*_1_ = 0.0371; τ_4_[Al(1)] = 0.91; O(1)---H(4), 1.853 Å. (iii) (HTriNOx^2–^)Al–OPh (**8**), *R*_1_ = 0.0612; τ_4_[Al(1)] = 0.94; O(1)---H(4), 1.891 Å. (iv) (HTriNOx^2–^)Ga–OPh (**9**), *R*_1_ = 0.0694; τ_4_[Ga(1)] = 0.93, O(1)---H(4), 1.902 Å.

Solid-state structures of complexes **4**, **6**, **8**, and **9** were obtained by single-crystal X-ray diffraction. Representations of the molecules are shown in Figure 1 and details regarding the collection and refinement of the data sets can be found in the Supporting Information. The complexes are all similar in structure, with the metal ion coordinated by four alkoxide ligands in a tetrahedral geometry (τ_4_ = 0.93–0.95). The average Al–O distance between the aluminum ion and the oxygen atoms of the TriNOx ligand in **4, 6**, and **8** is 1.777(3) Å. The similar set of Ga–O distances in **9** are longer (1.855(6) Å), as is expected with the increased size of the metal ion. In all the complexes, the M(OR)_4_ fragment sits at the top of the bonding pocket of the HTriNOx^2–^ ligand, with the protonated bridgehead N–H sitting at the bottom and pointing into the ligand. The N–H participates in hydrogen bonding with one of the oxygen atoms of a nitroxide group (O---H_ave_ = 1.862 Å for **4**, **6**, and **8**; O---H = 1.902 Å for **9**). The average N–O distance across all four complexes is 1.45 Å, which is in the range of analogous metrics observed in other metal complexes of the TriNOx ligand.^23−29^ The Al–O bond distances for the Al–O^*t*^Bu (1.7240(10) Å for **4**), Al–OBn (1.7446(8) Å for **6**), and Al–OPh (1.7453(12) Å for **8**) interactions are all in the range of the other structurally characterized terminal Al–OR bonds of their respective types.^30−32^ Similarly, at 1.852(4) Å the Ga–O bond distance for the Ga–OPh interaction in **9** is in the range of other structurally characterized terminal Ga–OPh bonds.^33^

**Figure 2:**
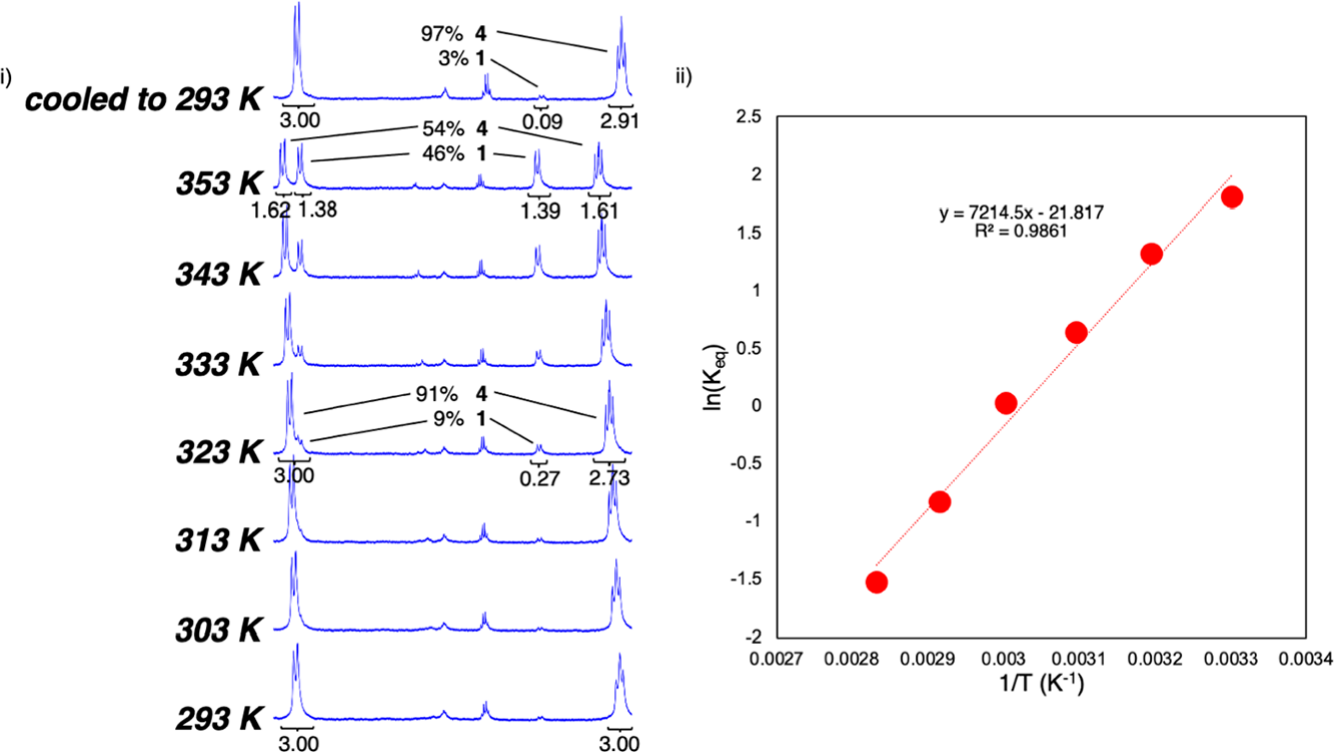
(i) Diastereotopic proton region of the ^1^H NMR spectra of the **4** complex over the temperature range 293–353 K; (ii) dependence of the ln(*K*_eq_) on temperature for the reaction **1** + *t-*BuOH ⇄ **4**.

The VT-NMR spectra of **4** were collected over the 293–353 K temperature range and demonstrate the reversibility of the alcohol addition reaction (Figure 2i). As the temperature of a sample of **4** is increased from 293 K, **1** and *t-*BuOH are formed at the expense of **4**. By 323 K, the **1**:**4** ratio is 0.09:0.91, determined via a comparison of the integrations of the bridgehead protons of the two metal complexes. Increasing the temperature to 353 K further increases the **1**:**4** ratio to 0.46:0.54. When returned to room temperature, the liberated *t-*BuOH adds to the (TriNOx^3–^)Al complex to reform the alcohol adduct **4**, giving a final **1**:**4** ratio of 0.03:0.97. The reaction is clean with **4**, **1**, and *t-*BuOH being the only species observed in the spectra over the temperature range investigated and with no noticeable generation of free TriNOxH_3_.

The enthalpy and entropy of the forward **1** + *t-*BuOH ⇄ **4** reaction were determined from the temperature dependence of the equilibrium constant for the K_eq_ for the reaction at various temperatures (Figure 2ii). The reaction is exothermic with a Δ*H* of −225 kJ/mol, which agrees with the VT-NMR experiment of **4**, which showed increasing amounts of **1** as T increases. The Δ*S* is large (−181 J/mol K), which we attribute to the forward reaction generating the more ordered (HTriNOx^2–^)Al–O^*t*^Bu adduct. Additionally, the creation of charge separation in the (HTriNOx^2–^)Al–O^*t*^Bu complex would also be expected to result in more organization of the solvent and hence a large negative entropy.

The VT-NMR spectra of both **6** and **8** were also collected (see the Supporting Information). Complex **6** behaves similarly to **4**, although the **1**:**6** ratio is smaller relative to the **1**:**4** ratio across every temperature examined with a final **1**:**6** ratio of 0.11:0.89 at 353 K. On return to room temperature, **6** is fully reformed with no trace of **1**. The phenoxide complex **8** is stable in solution, with no appearance of **1** across the 293–353 K temperature range. The expected Al–O bond strengths in the three complexes **4**, **6**, and **8** should be Al–OPh < Al–OBn < Al–O^*t*^Bu, as supported by the Al–O bond lengths observed in the solid-state structures. The observed thermal stabilities of the complexes as gauged by the VT-NMR experiments thus suggest that it is a competition between the Al–OR and H–OR bond strengths that determines the equilibrium.

We further explored these concepts by carrying out the reaction of **1** and **2** with a broader range of alcohols. With the exception of *t-*BuOH, **1** reacts to completion to give the (HTriNOx^2–^)Al–OR products with the majority of the alcohols we studied, and we highlight the reaction with both *i*-PrOH (Figure S19) and 9-fluorenemethanol (Figure S20) as specific examples. The position of the **1** + *t-*BuOH ⇄ **4** is solvent-dependent, with equilibrium lying further toward **4** in C_6_D_6_ relative to CDCl_3_ (see Figures S17 and S18). Interestingly, the **2** + *t-*BuOH ⇄ **5** equilibrium seems less solvent-dependent, with a conversion of ∼20% in either C_6_D_6_ or CDCl_3_.

The reactions with **1** give higher conversions in comparison to the reaction of the same alcohol with **2** across the range of alcohols studied. We attribute this difference to the bonding preferences for aluminum versus gallium. The RO^–^ ligands in the (HTriNOx^2–^)M–OR complexes are classified as hard according to Pearson’s theory of hard/soft acids and bases;^34,35^ as such, a given alkoxide should form a stronger bond with the harder aluminum ion relative to with the softer gallium ion, resulting in the (HTriNOx^2–^)Al–OR products being favored over their (HTriNOx^2–^)Ga–OR counterparts. To support this reasoning, we investigated the 1:1 reaction of **1** and **2** with *t*-butylmercaptan (*t-*BuSH, p*K*_a_ = 17.9), which would incorporate the softer *^t^*BuS^–^ anion in the presumptive (HTriNOx^2–^)M–S^*t*^Bu products (Figure S30). In the case of **1**, no adduct product is observed in the ^1^H NMR spectra of its reaction with *t-*BuSH after 24 h, although the resonances for **1** are all broadened in the presence of the thiol.^36^ Conversely, the reaction between the *t-*BuSH and **2** gives (HTriNOx^2–^)Ga–S^*t*^Bu in 82% conversion under identical reaction conditions.

**Figure 3:**
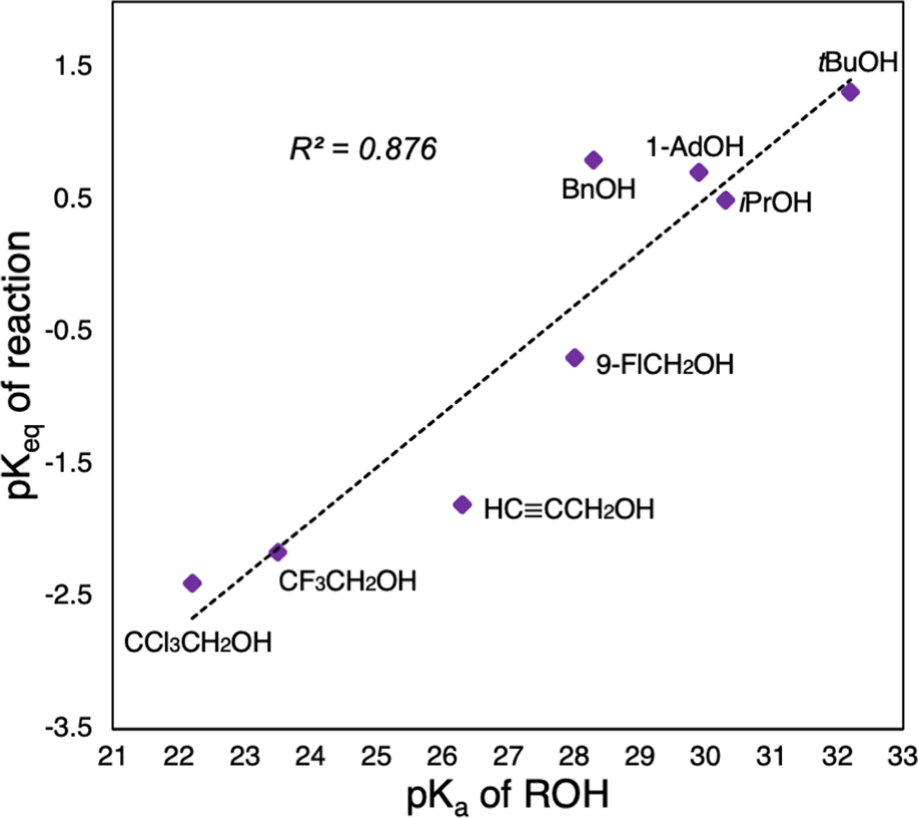
Plot of the reaction p*K*_eq_ versus ROH p*K*_a_ for the 1:1 reaction of (TriNOx^3–^)Ga (**2**) with various alcohols in CDCl_3_.

The full data for the gallium series shows a correlation between the p*K*_a_ of the alcohol^37^ and the reaction *K*_eq_, such that more acidic alcohols favor the formation of the (HTriNOx^2–^)Ga–OR products (Table 1, Figure 3). We would expect to see a similar trend for the (HTriNOx^2–^)Al–OR complexes, but in these cases, the majority of the reactions go to completion. There is no correlation between the reaction *K*_eq_ and size of the alcohol as judged by the A-value of the alkoxide ligand (see the Supporting Information). However, there is clearly an upper limit to the size of alkoxide that can be accommodated, since neither **1** or **2** react with 2,4,6-tri-*tert*-butylphenol even though the alcohol p*K*_a_ (22.8) would suggest a quantitative reaction in both cases.

**Table 1:**
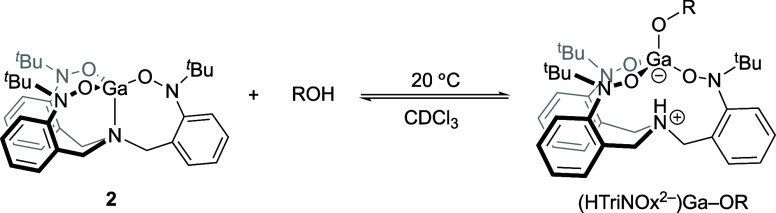
Percent Conversion and *K*_eq_ Values of Reactions of Various Alcohols with Complex **2**

entry	R	p*K*_a_^a^	% conversion^c^	*K*_eq_^d^
1	*^t^*Bu	32.2	21	0.049^e^
2	*^i^*Pr	30.3	42	0.32
3	1-adamantyl	29.9^b^	36	0.20
4	Bn	28.3^b^	38	0.16^e^
5	9-MeFl	28.0^b^	84	5.0
6	HCCCH_2_	26.3^b^	93	64
7	CF_3_CH_2_	23.5	91	150
8	CCl_3_CH_2_	22.2^b^	98	250

a In DMSO as reported in Hans Reich’s Bordwell p*K*_a_ table.^38^

b Approximated using G4. See the Supporting Information for details.

c Determined from the ratio of **2**:(HTriNOx^2–^)Ga–OR in the ^1^H NMR spectra of a 1:1 mixture of **2** and ROH.

d Calculated using concentrations of each species determined from integration of the ^1^H NMR spectra of a mixture of a 1:1 mixture of **2**:ROH against internal standard.

e Value determined in C_6_D_6_.

### Kinetic Analysis and Mechanistic Considerations

**Figure 4:**
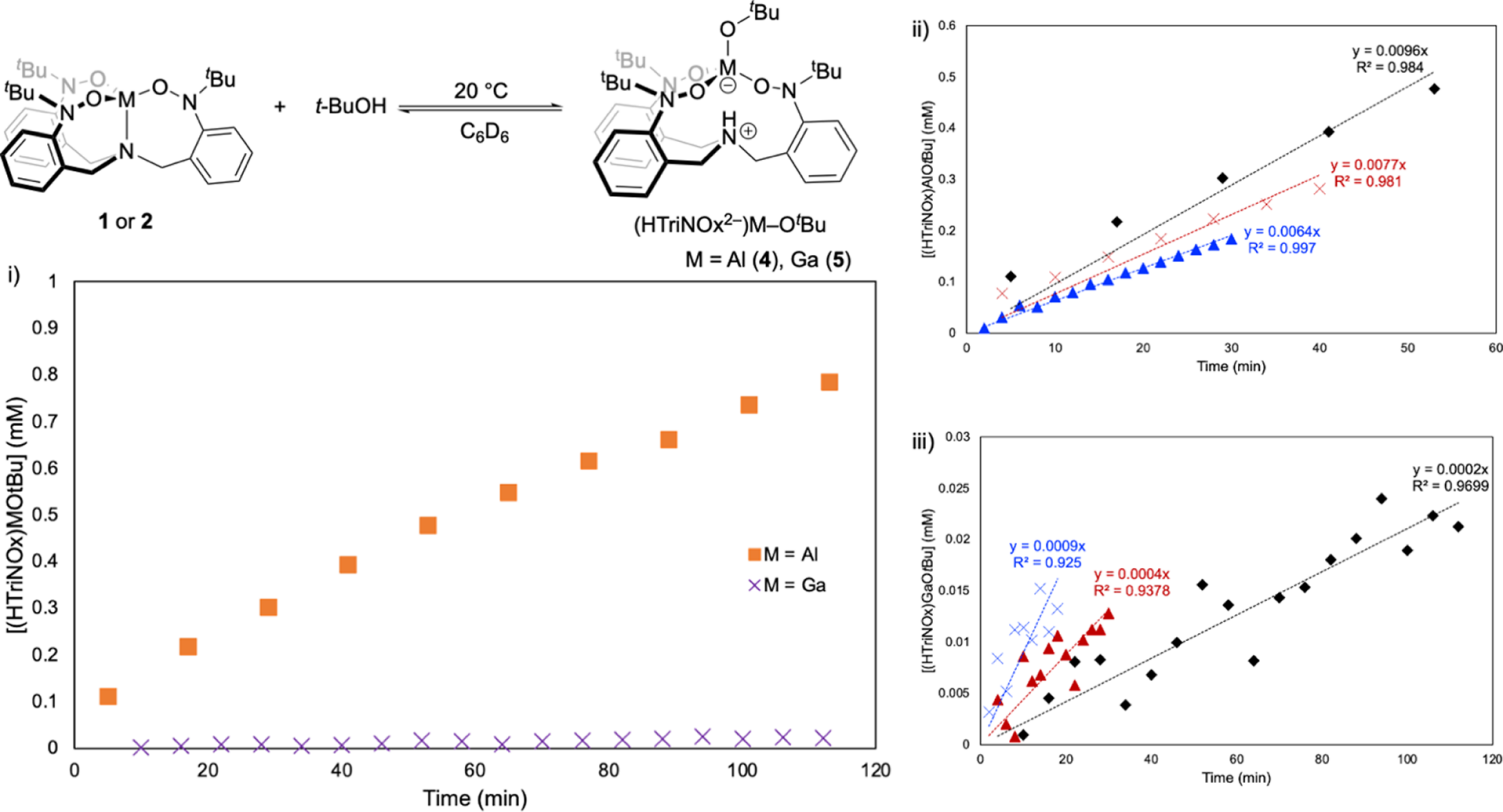
Reaction of **1** and **2** with *t*-BuOH in C_6_D_6_ at 20 °C: (i) Concentration of products over time for the two reactions. (ii) Initial rate data for the reaction of **1** with *t*-BuOH. (iii) Initial rate data for the reaction of **2** with *t*-BuOH. Replicate trials are represented by blue, black, and red lines.

Our initial observations on the reactivity of the (TriNOx^3–^)M complexes with alcohols suggested that the kinetic parameters of the reactions are also influenced by the identities of both the metal ion and the reacting alcohol. To more fully explore these observations, we monitored the conversion over time for the reaction of **1** and **2** with *t-*BuOH and *i-*PrOH to give the (HTriNOx^2–^)M–OR alcohol adducts. The reaction of *t-*BuOH proceeds with a rate constant an order of magnitude greater with **1** (*k* = 0.008 ± 0.002 min^–1^) relative to its reaction with **2** (0.0005 ± 0.0004 min^–1^) (Figure 4). A similar difference in rate was observed in the reactions of *i-*PrOH with **1** and **2** (see Supporting Information). In this case, *i-*PrOH reacts with **1** faster than our experimental capabilities to accurately determine a reaction rate, although our data suggests a rate constant of *k* ∼ 0.4 min^–1^.^39^ We were able to determine the rate constant for the reaction of **2** with *i-*PrOH (*k* = 0.009 ± 0.002 min^–1^), which when directly compared to the value for the reaction between **2** and *t*-BuOH suggests that more acidic alcohols result in faster reaction rates. The reactions between MeOH with **1** and **2** are both too fast to extract reliable rate constants, although the data clearly shows that the reaction with **1** is significantly faster than that with **2** and as predicted based on its lower p*K*_a_ is the fastest reacting alcohol studied for either metal complex.

**Scheme 4:**
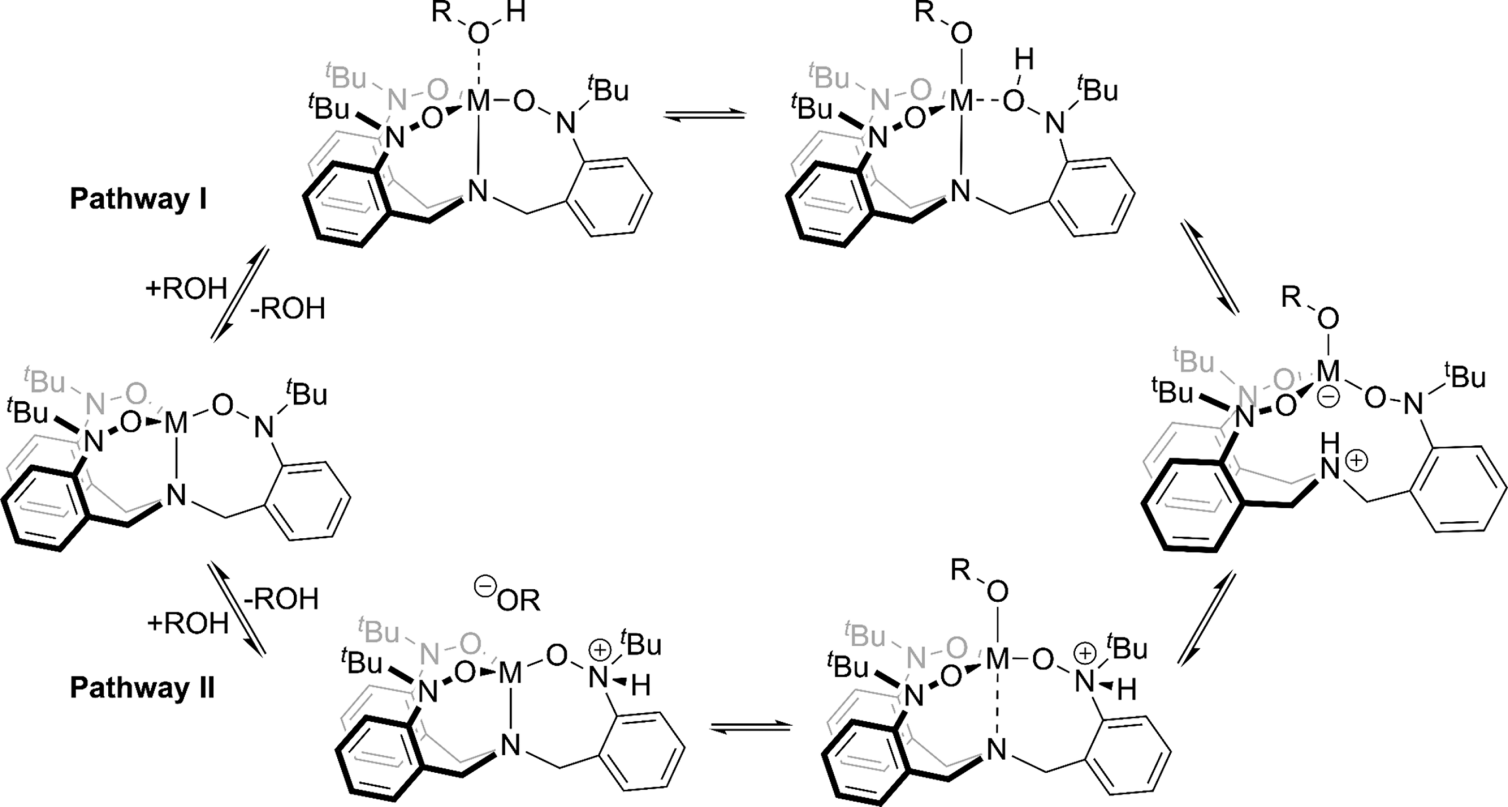
Mechanistic Proposals for the Reaction of **1** (M = Al) and **2** (M = Ga) with Alcohol to Give (HTriNOx^2–^)M–OR

We make the following mechanistic proposals based on this kinetic investigation (Scheme 4). First, we think that it is unlikely that the reaction proceeds through direct interaction between the M–N bond of the (TriNOx^3–^)M complexes with the O–H bond of the alcohol given that the M–N bond sits within the ligand pocket. Instead, we propose that the reaction first involves the formation of a Lewis acid–base adduct between the (TriNOx^3–^)M complex and alcohol (Pathway I). This is expected to be less favorable for **2** relative to **1** given their relative Lewis acidities (vide supra) which results in faster reaction rates for **1** versus **2** for any given alcohol. Upon coordination with the metal, the alcohol proton becomes more acidic and transfers to an oxygen atom of one of the N–O arms of the TriNOx ligand, installing the alkoxo ligand and protonating the ligand backbone. We expect this step to be rate-limiting and dependent on the p*K*_a_ of the reacting alcohol. A second proton transfer to the basal nitrogen commensurate with breaking the M–N bond generates the (HTriNOx^2–^)M–OR products.

We have also considered a mechanism where the alcohol protonates the TriNOx ligand to generate the ion pair [(HTriNOx^2–^)M][^−^OR] as the initial step of the reaction (Pathway II). It is not clear which site on the TriNOx ligand would be protonated, although our previous study on the reactivity of **1-py** with MeOTf suggests that the nitrogen atoms of the nitroxide arms are the most basic sites in the (TriNOx^3–^)Al complex.^15^ After this protonation step, coordination of the alkoxide anion to the metal ion installs the alkoxo ligand, which we expect to simultaneously weaken the M–N interaction. The final step involves proton transfer to the basal nitrogen commensurate with fully breaking the M–N bond to give the (HTriNOx^2–^)M–OR products. The rate dependence on the alcohol p*K*_a_ is highlighted in this mechanistic pathway, although it is less obvious why **1** would react an order of magnitude faster than **2**. However, Pathway II offers a reasonable route to explain how the (HTriNOx^2–^)Ga–OR complexes are formed without precoordination of the alcohol to the gallium ion in **2**, which we have evaluated as lacking Lewis acidity. This is also in agreement with the chemistry reported by the Greb group, who showed enhanced ELC between their calix[4]pyrrolato gallate complex with *i*-PrOH with preprotonation of the ligand framework.^13^ At this point, it is unclear whether one or both pathways are operative, and the specific nature of the proton transfer steps involved in either pathway is not fully elucidated. With these qualifiers, it is tempting to hypothesize that the two complexes **1** and **2** may proceed through different pathways, especially given the difference in rate constants between the complexes. We are currently investigating the mechanistic details more fully.

## Experimental Section

### Physical Measurements

All NMR spectra were recorded using a Bruker 400 MHz spectrometer (399.78 MHz for ^1^H, 100.52 MHz for ^13^C) at ambient temperature unless otherwise specified. Chemical shifts were referenced to residual solvent. s = singlet, bs = broad singlet, d = doublet, t = triplet, dd = doublet of doublets, m = multiplet. CHN analyses were performed at the CENTC Elemental Analysis Facility at the University of Rochester (for **4**, **6**, **8**, and **9**) or at the Midwest Microlab (for **2** and **3**).

### Safety Statement

No uncommon hazards are noted.

### Preparation of Compounds

All reactions and manipulations were performed under an inert atmosphere (N_2_) using standard Schlenk techniques or in a Vacuum Atmospheres, Inc. NextGen glovebox equipped with oxygen and moisture purifier systems. Glassware was dried overnight at 160 °C before use. C_6_D_6_, CDCl_3_, THF-*d*8, and pyridine-*d*5 were degassed and stored over 4 Å molecular sieves prior to use. Tetrahydrofuran, toluene, dichloromethane, hexane, and pentane were sparged for 20 min with dry argon and dried using a commercial two-column solvent purification system comprising of two columns packed with neutral alumina (for tetrahydrofuran and dichloromethane) or Q5 reactant then neutral alumina (for hexanes, toluene, and pentane). Anhydrous benzene and pyridine were further dried over 4 Å molecular sieves prior to use. The (TriNOx)H_3_ ligand precursor,^23^ [M(NMe_2_)_3_]_2_ (M = Al, Ga),^40^ and [In(NEt_2_)_3_]_2_^41^ starting materials were prepared according to literature procedures. All other reagents were purchased from commercial sources and used as received.

### General Protocol for the Synthesis of (TriNOx^3–^)M (**1**, M = Al; **2**, M = Ga)

TriNOxH_3_ (0.50 g, 0.91 mmol) was loaded into a round-bottom Schlenk flask equipped with a stir bar and dissolved in toluene (30 mL). [M(NMe_2_)_3_]_2_ (0.41 mmol) was then added to the reaction, and the flask was sealed, removed from the glovebox, and heated at 50 °C. After 12 h, the reaction was removed from heat, cooled to room temperature, and brought back into the glovebox where volatiles were removed from the reaction mixture under reduced pressure. The resulting material was triturated with pentane (3 × 10 mL) to give **1** or **2** as off-white solids.

Characterization data for **1**: Yield = 0.42 g, 0.73 mmol (80%).^16^^1^H NMR (CDCl_3_): δ 7.57 (d, *J* = 8 Hz, 3H), 7.27 (m, 6H), 7.06 (t, *J* = 7 Hz, 3H), 4.47 (d, *J* = 12 Hz, 3H, NC*H*_2_), 3.05 (d, *J* = 12 Hz, 3H, NC*H*_2_), 1.27 (s, 27H, C(C*H*_3_)_3_). ^13^C{^1^H} NMR (CDCl_3_): 152.4, 133.0, 132.1, 129.1, 128.2, 124.1, 61.5, 58.2, 27.8. The elemental purity of **1** has previously been confirmed as the **1-py** adduct.^15^

Characterization data for **2**. Yield: 0.39 g, 0.64 mmol (70%).^16^^1^H NMR (C_6_D_6_): δ 7.63 (d, *J* = 8 Hz, 3H), 7.04 (m, 6H), 6.90 (t, *J* = 7 Hz, 3H), 4.87 (d, *J* = 12 Hz, 3H, NC*H*_2_), 2.84 (d, *J* = 12 Hz, 3H, NC*H*_2_), 1.40 (s, 27H, C(C*H*_3_)_3_). ^13^C{^1^H} NMR (C_6_D_6_): δ 152.6, 133.1, 132.2, 129.4, 124.9, 124.4, 62.2, 58.2, 27.7. Anal. Calcd for C_33_H_45_GaN_4_O_3_: C, 64.40; H, 7.37; N, 9.10. Found: C, 63.77; H, 7.46; N, 9.01. Crystals suitable for X-ray diffraction were obtained from a saturated THF solution layered with hexane at −25 °C.

### Synthesis of (TriNOx^3–^)In (**3**)

[In(NEt_2_)_3_]_2_ (0.21 g, 0.32 mmol) was added to a round-bottom Schlenk flask equipped with a stir bar and dissolved in toluene (∼25 mL). TriNOxH_3_ (0.35 g, 0.64 mmol) was separately dissolved in toluene (∼25 mL) and transferred to the Schlenk flask. The reaction was allowed to stir at room temperature for 12 h after which volatiles were removed from the heterogeneous reaction mixture under reduced pressure. The crude reaction mixture was dissolved in boiling pyridine (∼20 mL), and the resulting solution was allowed to slowly cool to −25 °C. After 24 h, the resultant white powder was collected over a medium-porosity frit, washed with cold pyridine followed by hexane and then dried under vacuum to give **3** as a white powder. Yield: 0.12 g, 0.18 mmol (56%). ^1^H NMR (py-*d*5): δ 7.84 (d, *J* = 8 Hz, 3H), 7.41 (m, 6H), 7.24 (t, *J* = 7 Hz, 3H), 5.14 (d, *J* = 11 Hz, 3H, NC*H*_2_), 2.58 (d, *J* = 11 Hz, 3H, NC*H*_2_), 0.99 (s, 27H, C(C*H*_3_)_3_); ^13^C{^1^H} NMR (py-*d*5): δ 153.0, 134.2, 132.8, 129.0, 127.9, 124.9, 60.3, 59.7, 26.3. Anal. Calcd for C_33_H_45_InN_4_O_3_: C, 60.00; H, 6.87; N, 8.48. Found: C, 59.67; H, 6.70; N, 8.27.

### Synthesis of (HTriNOx^2–^)Al–O^*t*^Bu (**4**)

*tert*-Butanol (38.8 mg, 0.52 mmol) was dissolved in toluene (∼1 mL) and added to a stirring toluene (10 mL) solution of **1** (100 mg, 0.18 mmol) in a vial. The homogeneous mixture was allowed to stir at room temperature for 12 h, after which volatiles were removed from the reaction mixture via vacuum evaporation to give **3** as a white solid. Yield: 89 mg, 0.14 mmol (81% yield). ^1^H NMR (C_6_D_6_): δ 10.94 (bs, 1H), 7.82 (d, *J* = 8.0 Hz, 3H), 6.89 (t, J = 7.4, 3H), 6.70 (d, *J* = 6.4 Hz, 3H), 4.70 (d, *J* = 12.0 Hz, 3H), 2.24 (dd, *J*_1_ = 12.0 Hz, *J*_2_ = 10.0 Hz, 3H), 1.81 (s, 9H, OC(CH_3_)_3_), 1.00 (s, 27H, C(CH_3_)_3_).^42^^13^C{^1^H} NMR (C_6_D_6_): δ 154.3, 132.1, 131.7, 129.5, 126.1, 124.3, 67.8, 61.5, 57.1, 34.6, 26.7. Anal. Calcd for C_37_H_55_AlN_4_O_4_·(CH_2_Cl_2_)_1.5_: C, 59.80; H, 7.43; N, 7.25. Found: C, 59.90; H, 7.19; N, 7.42. Crystals suitable for X-ray diffraction were obtained from a saturated THF solution layered with hexane at −25 °C.

### Synthesis of (HTriNOx^2–^)Al–OBn (**6**)

Benzyl alcohol (19.0 mg, 0.176 mmol) was added as a solution in toluene (1 mL) to a stirring toluene (10 mL) solution of **1** (100 mg, 0.18 mmol) in a vial. The reaction was allowed to stir at room temperature for ∼12 h. Volatiles were subsequently removed from the reaction via vacuum evaporation to give **6** as a white powder. Yield: 100 mg, 0.15 mmol (82% yield). ^1^H NMR (C_6_D_6_): δ 11.03 (bs, 1H), 7.93 (d, *J* = 8 Hz, 2H), 7.85 (d, *J* = 8 Hz, 3H), 7.67 (m, 1H), 7.38 (t, *J* = 8 Hz, 3H), 6.90 (m, 5H), 6.71 (d, *J* = 8 Hz, 3H), 5.59 (d, *J* = 12 Hz, 1H), 5.51 (d, *J* = 12 Hz, 1H), 4.71 (d, *J* = 12 Hz, 3H), 2.25 (dd, *J*_1_ = 12 Hz, *J*_2_ = 8 Hz, 3H), 0.94 (s, 27H, C(C*H*_3_)_3_). ^13^C{^1^H} NMR (CDCl_3_): δ 153.3, 132.1, 131.1, 129.3, 129.0, 127.2, 126.4, 125.7, 125.0, 124.7, 65.2, 61.4, 57.5, 26.3. Anal. Calcd for C_40_H_53_AlN_4_O_4_·(CH_2_Cl_2_): C, 64.39; H, 7.11; N, 7.33. Found: C, 64.59; H, 7.10; N, 6.77. Crystals suitable for X-ray diffraction were obtained from a saturated THF solution layered with hexane at −25 °C.

### Synthesis of (HTriNOx^2–^)Al–OPh (**8**)

Phenol (16.5 mg, 0.175 mmol) was added as a solid to a toluene (10 mL) solution of **1** (100 mg, 0.175 mmol) stirring in a vial. The reaction was allowed to stir at room temperature for ∼12 h after which volatiles were subsequently removed from the reaction under reduced pressure to give **8** as a white powder. Yield: 100 mg, 0.15 mmol (82%). ^1^H NMR (CDCl_3_):^43^ δ 10.94 (bs, 1H), 7.66 (d, *J* = 8 Hz, 3H), 7.30 (t, *J* = 8 Hz, 3H), 7.05 (m, 7H), 6.96 (d, *J* = 8 Hz, 2H), 6.59 (t, *J* = 8 Hz, 2H), 4.95 (d, *J* = 12 Hz, 3H), 3.10 (dd, *J*_*1*_ = 12 Hz, *J*_*2*_ = 10 Hz, 3H), 0.64 (s, 27H, C(C*H*_3_)_3_). ^13^C{^1^H} NMR (CDCl_3_): δ 153.3, 132.2, 131.1, 129.4, 128.3, 125.6, 124.8, 121.7, 120.6, 116.3, 61.6, 57.5, 26.1. Anal. Calcd for C_39_H_51_AlN_4_O_4_·(CH_2_Cl_2_)_0.5_: C, 66.98; H, 7.26; N, 7.91. Found: C, 66.10; H, 7.28; N, 7.46. Crystals suitable for X-ray diffraction were obtained from a saturated THF solution layered with hexane at −25 °C.

### Synthesis of (HTriNOx^2–^)Ga–OPh (**9**)

Phenol (15.4 mg, 0.164 mmol) was added as a solid to a stirring toluene (∼10 mL) solution of **2** (100 mg, 0.164 mmol) in a vial. The reaction was allowed to stir at room temperature for ∼12 h after which volatiles were removed from the reaction under vacuum to give **9** as a white powder. Yield: 85 mg, 0.12 mmol (75%). ^1^H NMR (CDCl_3_):^43^ δ 10.91(bs, 1H, N*H*), 7.67 (d, *J* = 8 Hz, 3H), 7.30 (t, *J* = 8 Hz, 3H), 7.04 (m, 9H), 6.59 (t, *J* = 8 Hz, 2H), 4.95 (d, *J* = 12 Hz, 3H), 3.09 (dd, *J*_*1*_ = 12 Hz, *J*_*2*_ = 12 Hz, 3H), 0.66 (s, 27H, C(C*H*_3_)_3_). ^13^C{^1^H} NMR (CDCl_3_): δ 162.8, 153.1, 132.2, 130.9, 129.4, 128.4, 125.4, 124.8, 120.2, 116.3, 61.8, 57.5, 26.2. Anal. Calcd for C_39_H_51_GaN_4_O_4_·(CH_2_Cl_2_)_0.75_: C, 61.82; H, 6.72; N, 7.25. Found: C, 61.87; H, 6.66; N, 7.05. Crystals suitable for X-ray diffraction were obtained from a saturated THF solution layered with hexane at −25 °C.

## Conclusions

In summary, we have shown that the O–H bond of alcohols can be cleaved via an ELC pathway by the tripodal complexes (TriNOx^3–^)Al and (TriNOx^3–^)Ga. The thermodynamic and kinetic aspects of the reactions are both influenced by the identity of the metal, with **1** having higher reaction equilibrium constants and proceeding at a faster rate relative to **2** for any given alcohol. These parameters are also influenced by the p*K*_a_ of the alcohol, with more acidic alcohols reacting both to more completion and faster than their less acidic counterparts. We expect this knowledge to lay the groundwork for the ELC of other polar H–X bonds, an area we are actively exploring. Additionally, we are currently trying to better understand the differences in Lewis acidity between **1** and **2**, especially in how these differences result in divergent reactivity.

## Supporting Information Available

^1^H and ^13^C{^1^H} NMR spectra for all compounds, experimental protocols for the Guttmann, VT-NMR, van’t Hoff, and *K*_eq_ experiments, ^1^H NMR spectra used to determine the *K*_eq_ values, details regarding collection of the kinetic data, and details regarding the calculations of alcohol p*K*_a_ and R-group A-factors (PDF)
