# Supplementary material for: Reversible O–H Bond Activation by Tripodal tris(Nitroxide) Aluminum and Gallium Complexes
Source: Inorg Chem. 2024 Feb 22;63(9):4028–38. doi: 10.1021/acs.inorgchem.3c02902 (PMC10915791; doi:10.1021/acs.inorgchem.3c02902)
Supplement: Supplementary file 1 — ic3c02902_si_001.pdf [file ic3c02902_si_001.pdf]

Reversible O–H bond activation by tripodal tris(nitroxide) aluminum and gallium complexes.

Joseph S. Scott,[1] Mika L. Maenaga,[1] Audra J. Woodside,[1] Vivian W. Guo,[1] Alex R. Cheriel,[1] Michael R. Gau,[2] Paul R. Rablen,[1] Christopher R. Graves[1,]*

[1] Department of Chemistry & Biochemistry, Swarthmore College, 500 College Avenue, Swarthmore, PA 19081, United States

[2] Department of Chemistry, University of Pennsylvania, 231 South 34th Street, Philadelphia, PA 19104, United States

Electronic Supplementary Information

Author contribution statement S3

Figure S1. ^{1}H NMR spectrum of (TriNOx^{3-})Al (**1**) S4

Figure S2. ^{13}C{^{1}H} spectrum of (TriNOx^{3-})Al (**1**) S5

Figure S3. ^{1}H NMR spectrum of (TriNOx^{3-})Ga (**2**) S6

Figure S4. ^{13}C{^{1}H} NMR spectrum of (TriNOx^{3-})Ga (**2**) S7

Figure S5. ^{1}H NMR spectrum of (TriNOx^{3-})In (**3**) S8

Figure S6. ^{13}C{^{1}H} NMR spectrum of (TriNOx^{3-})In (**3**) S9

Figure S7. ^{1}H NMR spectrum of (HTriNOx^{2-})Al–O*^{t}*Bu (**4**) S10

Figure S8. ^{13}C{^{1}H} NMR spectrum of (HTriNOx^{2-})Al–O*^{t}*Bu (**4**) S11

Figure S9. ^{1}H NMR spectrum of (HTriNOx^{2-})Al–OBn (**6**) S12

Figure S10. ^{13}C{^{1}H} NMR spectrum of (HTriNOx^{2-})Al–OBn (**6**) S13

Figure S11. ^{1}H NMR spectrum of (HTriNOx^{2-})Al–OPh (**8**) S14

Figure S12. ^{13}C{^{1}H} NMR spectrum of (HTriNOx^{2-})Al–OPh (**8**) S15

Figure S13. ^{1}H NMR spectrum of (HTriNOx^{2-})Ga–OPh (**9**) S16

Figure S14. ^{13}C{^{1}H} NMR spectrum of (HTriNOx^{2-})Ga–OPh (**9**) S17

Protocol for determination of Lewis acid acceptor numbers for **1** and **2** S18

Figure S15. Stacked plot of the $^{31}P\{^1H\}$ NMR spectra of the mixtures of Et_3PO (i) and Et_3PS (ii) experiments run to evaluate the Lewis acidity of complexes **1** and **2**. S18

Protocol for the VT-NMR studies of complexes **4**, **6**, and **8** S19

Figure S16. Diastereotopic proton region of the ^1H NMR spectra of the **4**, **6**, and **8** complexes over the temperature range 293–353 K. S19

Protocol for the van't Hoff experiment of **1** + *t*-BuOH. S20

Protocol for determining the K_{eq} values for the reactions of **1** and **2** with various alcohols S20

Figure S17. ^1H NMR spectrum of a 1:1 mixture of **1**:*t*-BuOH in C_6D_6 S21

Figure S18. ^1H NMR spectrum of a 1:1 mixture of **1**:*t*-BuOH in $CDCl_3$ S22

Figure S19. ^1H NMR spectrum of a 1:1 mixture of **1**:*i*-PrOH. S23

Figure S20. ^1H NMR spectrum of a 1:1 mixture of **1**:9-fluorenemethanol. S24

Figure S21. ^1H NMR spectrum of a 1:1 mixture of **2**:*t*-BuOH in C_6D_6 S25

Figure S22. ^1H NMR spectrum of a 1:1 mixture of **2**:*t*-BuOH in $CDCl_3$ S26

Figure S23. ^1H NMR spectrum of a 1:1 mixture of **2**:*i*-PrOH S27

Figure S24. ^1H NMR spectrum of a 1:1 mixture of **2**:1-adamantanol. S28

Figure S25. ^1H NMR spectrum of a 1:1 mixture of **2**:BnOH. S29

Figure S26. ^1H NMR spectrum of a 1:1 mixture of **2**:9-fluorenemethanol. S30

Figure S27. ^1H NMR spectrum of a 1:1 mixture of **2**:propargyl alcohol. S31

Figure S28. ^1H NMR spectrum of a 1:1 mixture of **2**:CF_3CH_2OH. S32

Figure S29. ^1H NMR spectrum of a 1:1 mixture of **2**:CCl_3CH_2OH. S33

Figure S30. ^1H NMR spectra of the reaction of *t*-BuSH with **1** and **2** S34

Protocol for kinetic analysis of the reactions of **1** and **2** with alcohols S35

Figure S31. Plot showing the concentration of products over time for the reaction of **1** and **2** with *i*-PrOH S35

Figure S32. Initial rate data for the reaction of **2** with *i*-PrOH. S35

Procedure for the calculations to give the predicted-pK_a of alcohols in DMSO. S36

Table S1. Raw calculated Gibbs standard free energies at 298 K of alcohols and their corresponding alkoxides. S37

Table S2. Calculated standard Gibbs free energies of deprotonation (ΔG_{deprot}) in DMSO for alcohols and the manipulation of that data to give predicted pK_a values for alcohols. S38

Figure S33. Correlation plot between the G4-calculated versus experimental determined pK_a values for alcohols. S39

Procedure for the calculations to determine the A-values for the alcohol R groups. S40

Table S3. Calculated standard Gibbs free energies at 298 K of the equatorial ($\Delta G^0_{equatorial}$) and axial (ΔG^0_{axial}) conformers of R-substituted cyclohexanes and the calculated A-values for the R groups. S40

Figure S34. Plot of the pK_{eq} of the reaction of **2** with alcohol versus the A-value of the alcohol R group. S41

Author contribution statement. J.S.S. contributed to the synthesis of **1** and **2** and carried out the Gutmann-Beckett experiments; carried out the reactivity studies between **1** and **2** with alcohols; developed the syntheses and contributed to the characterization of **4**, **6**, **8** and **9**, including all VT-NMR experiments; carried out all K_{eq} and kinetic experiments; conducted calculations to determine alcohol pK_a values and R group A-values; drafted and revised the manuscript. M.L.M. contributed to the synthesis of **1** and **2**; carried out preliminary reactivity studies of **1** and **2** with alcohols; contributed to the synthesis and characterization of **4**, **8**, and **9**; contributed to the calculations to determine alcohol pK_a values; proposed mechanistic considerations. A.J.W. contributed to the synthesis of **1-3**; carried out preliminary reactivity studies of **1** and **2** with alcohols. V. W. G. contributed to the synthesis of **1** and **2**; contributed to the synthesis and characterization of **4**, **6**, **8** and **9**. A.R.C. contributed to the synthesis of **1** and **2**; helped in the drafting and revising of the manuscript. M. R. G. conducted x-ray crystallography. P. R. R. supervised the calculations to determine alcohol pK_a values and R group A-values. C.R.G. supervised and administered all aspects of the project and drafted and revised the manuscript.

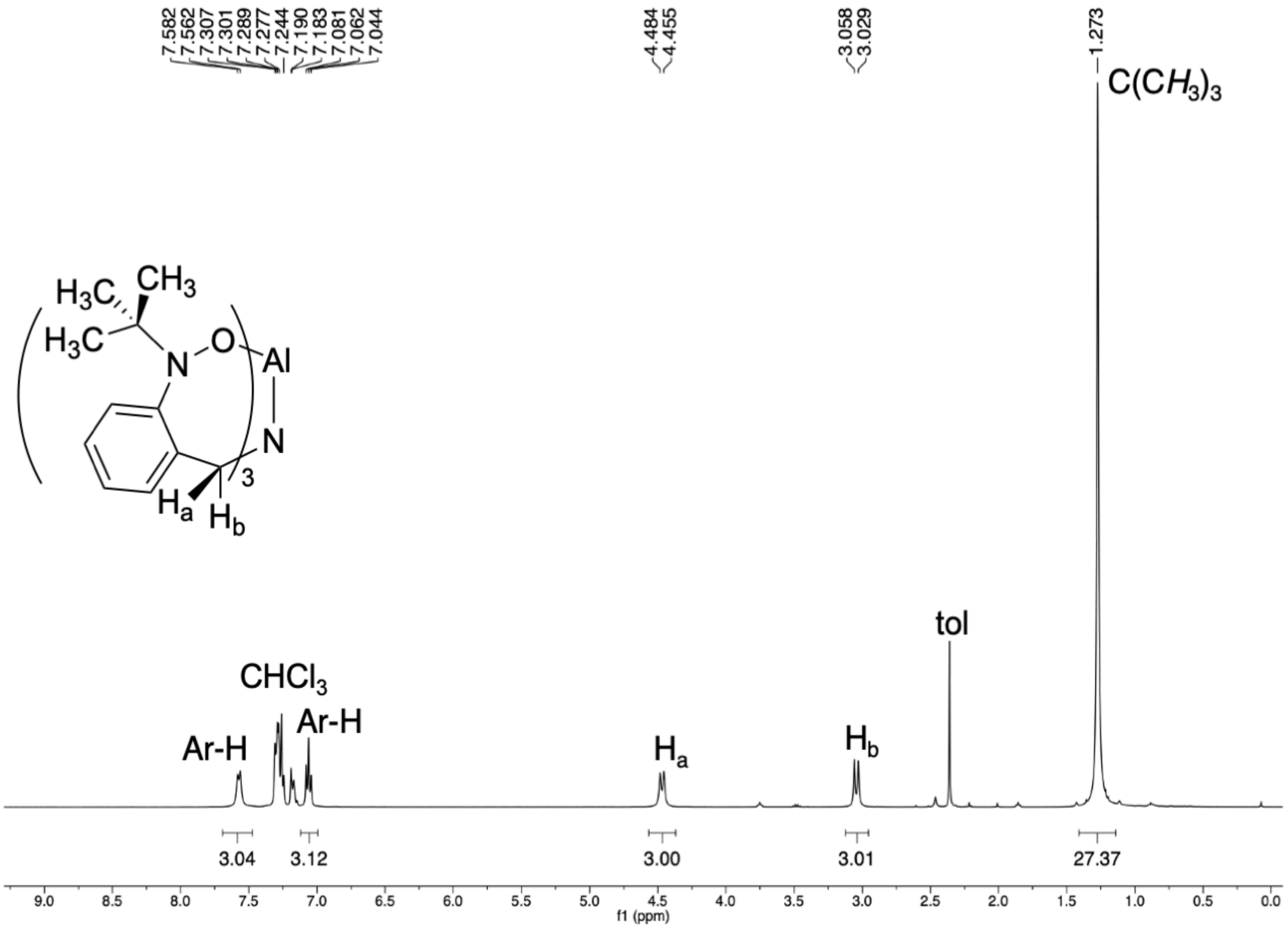

Figure S1. ^{1}H NMR spectrum of (TriNOx^{3-})Al (**1**) in $CDCl_3$.

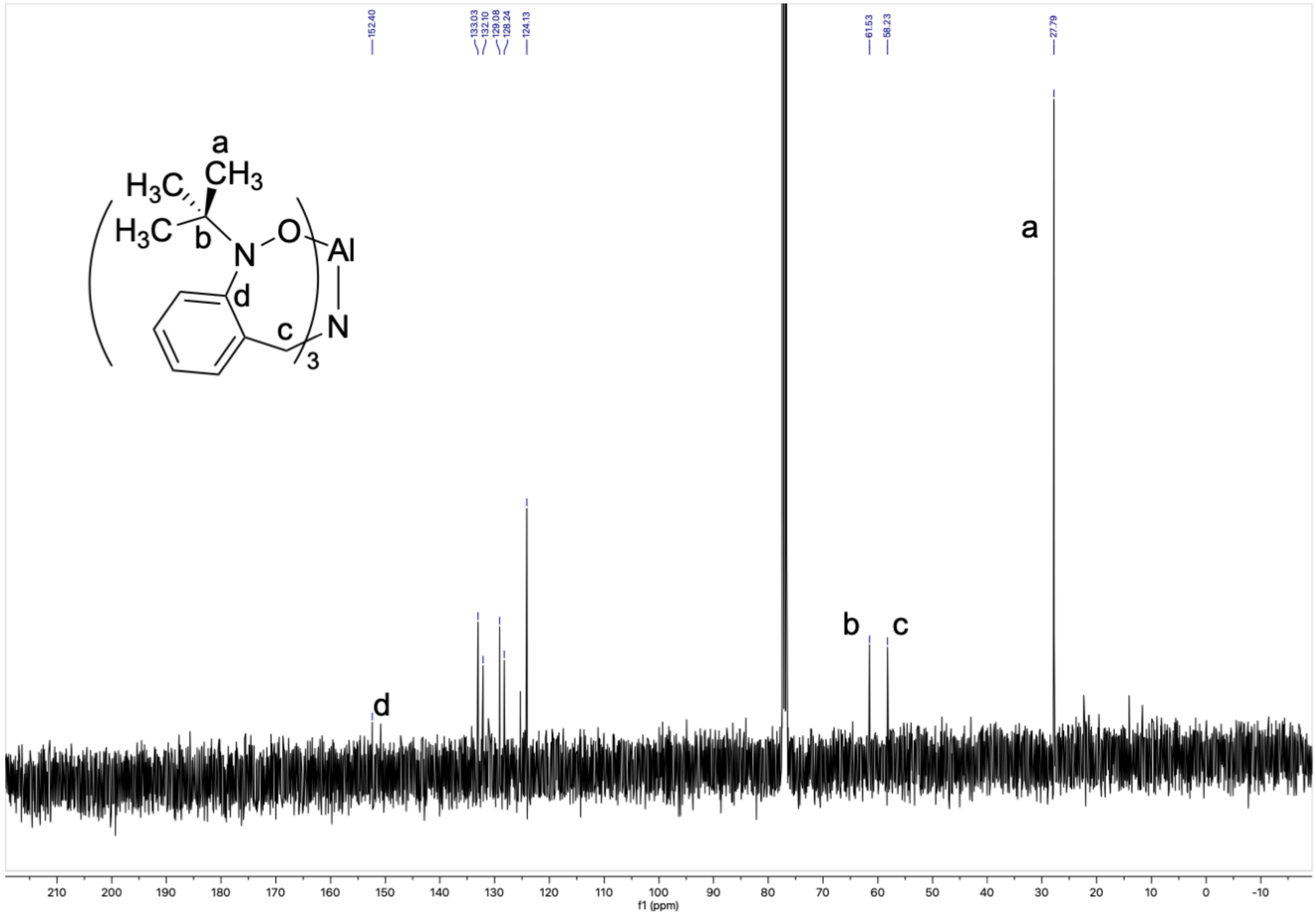

Figure S2. $^{13}C\{^1H\}$ NMR spectrum of $(TriNOx^{3-})Al$ (**1**) in $CDCl_3$.

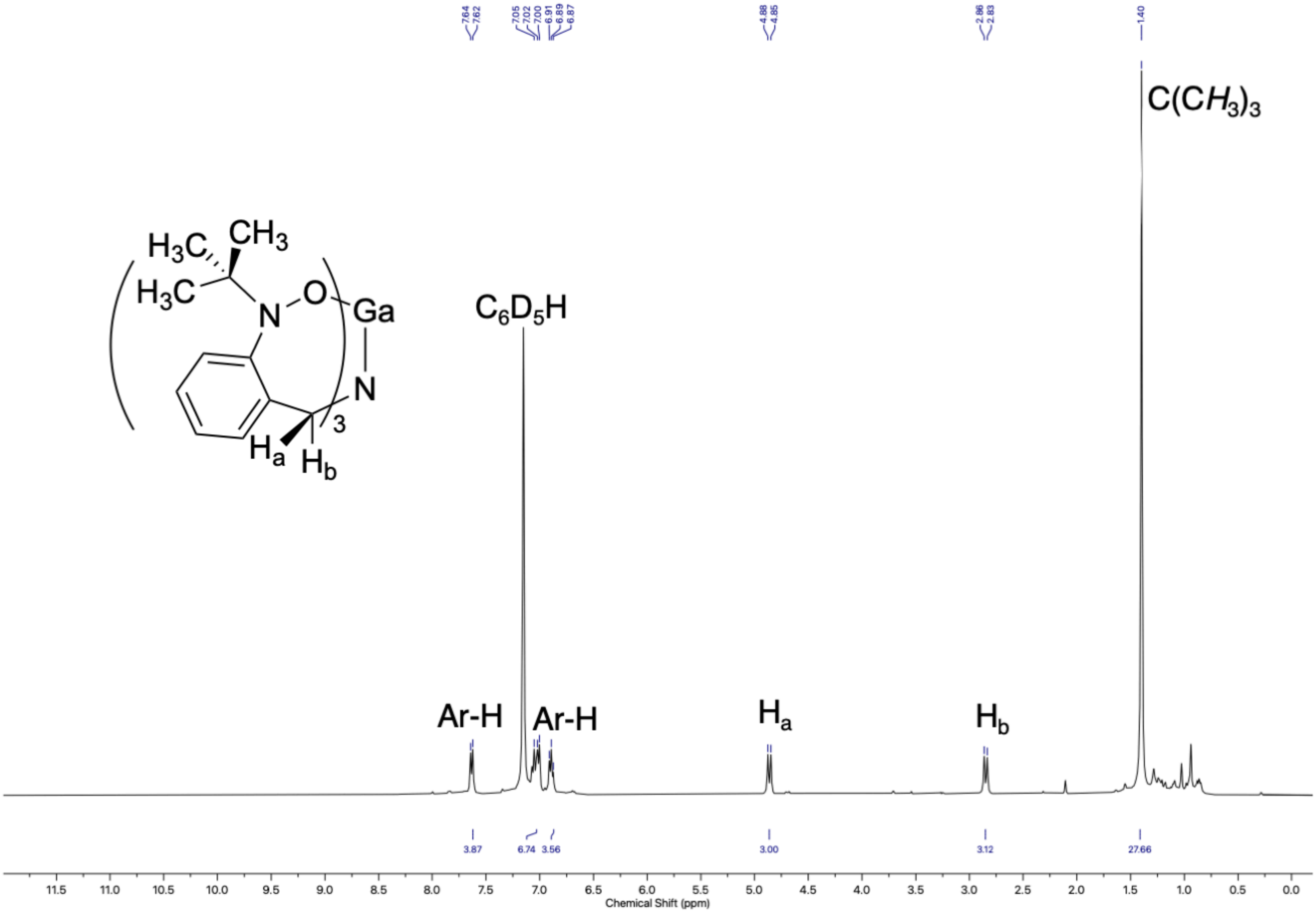

Figure S3. ^{1}H NMR spectrum of (TriNOx^{3-})Ga (**2**) in C_6D_6.

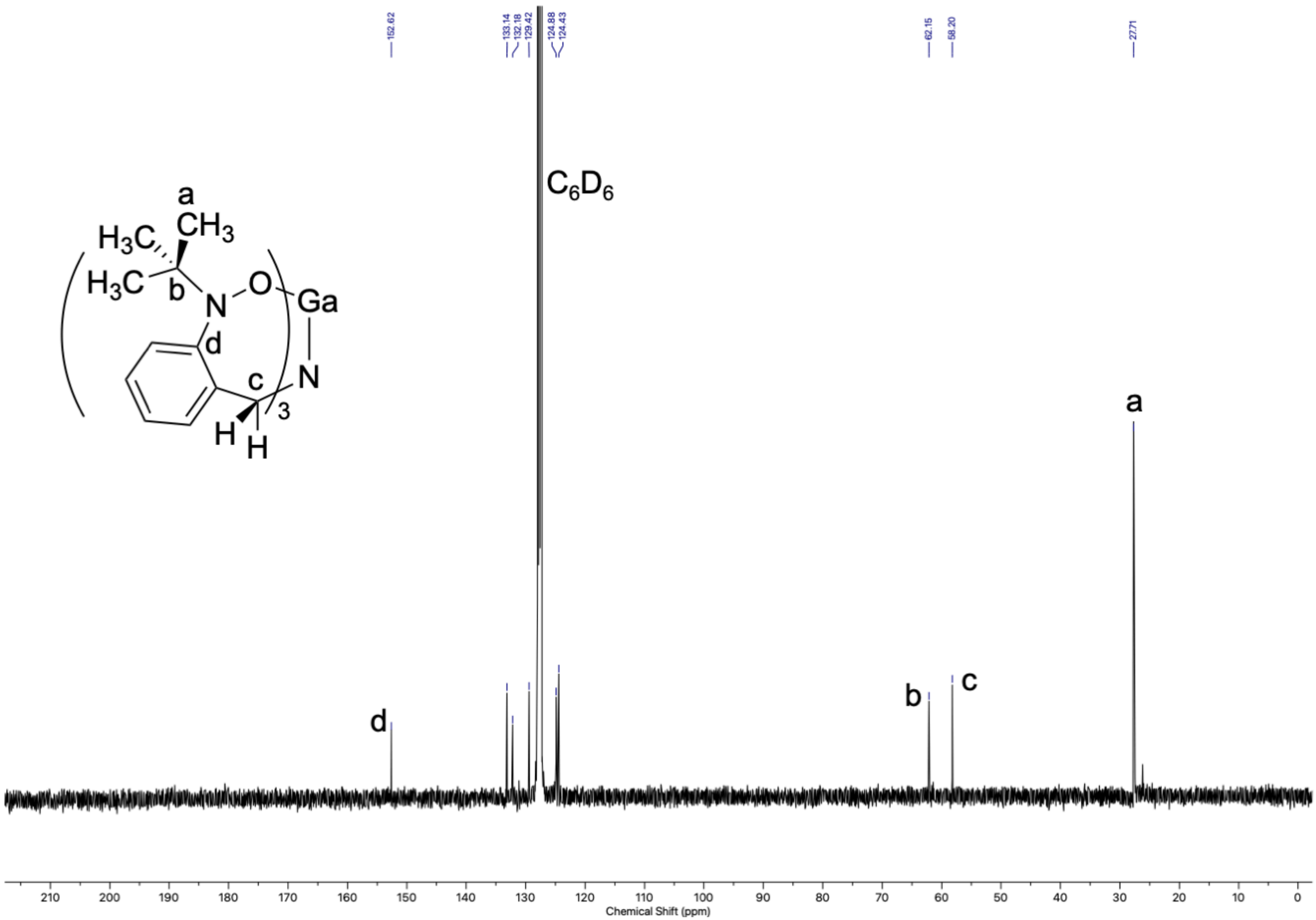

Figure S4. $^{13}C\{^{1}H\}$ NMR spectrum of (TriNOx^{3-})Ga (**2**) in C_6D_6.

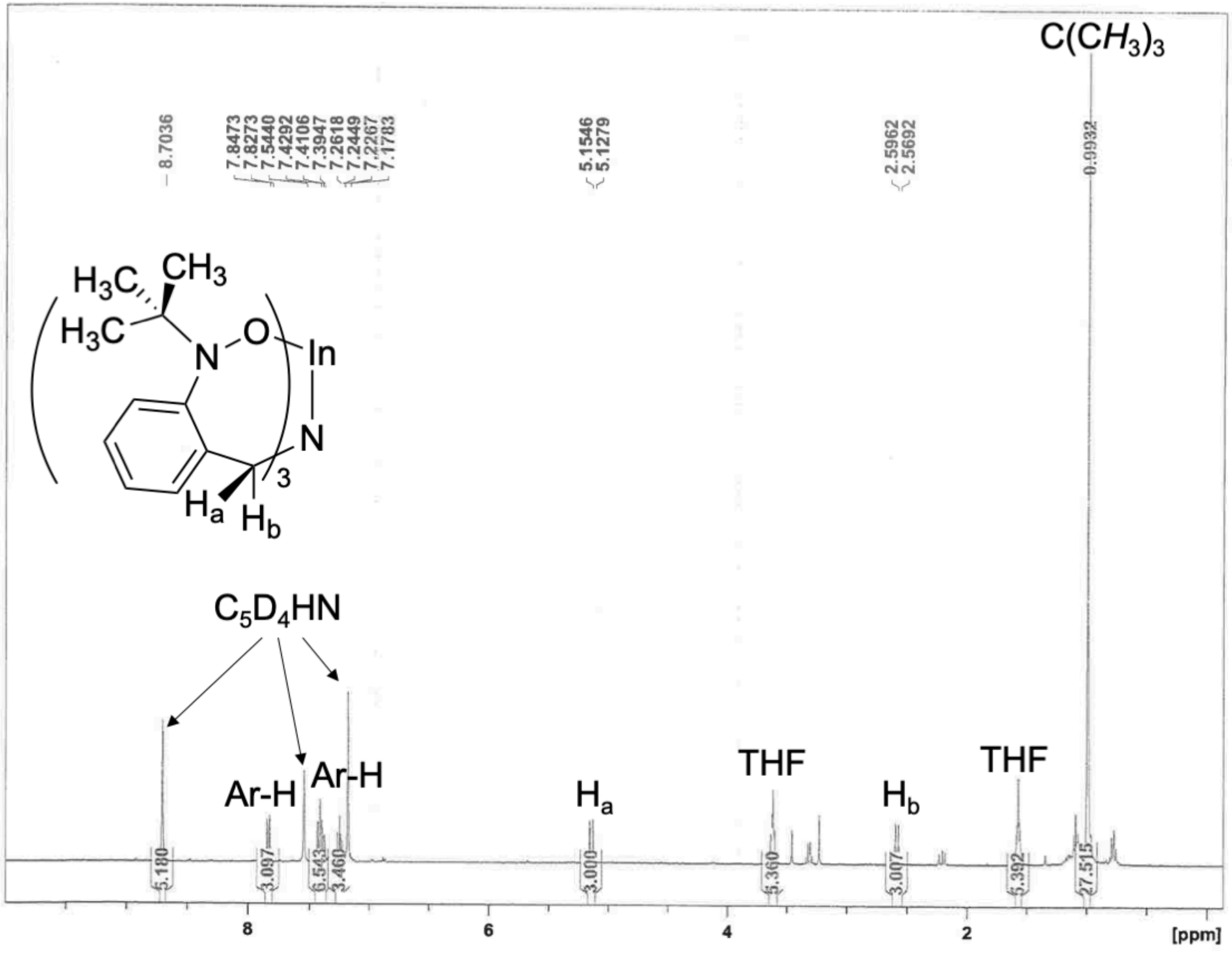

Figure S5. ^{1}H NMR spectrum of (TriNOx^{3-})In (**3**) in pyridine-*d*5.

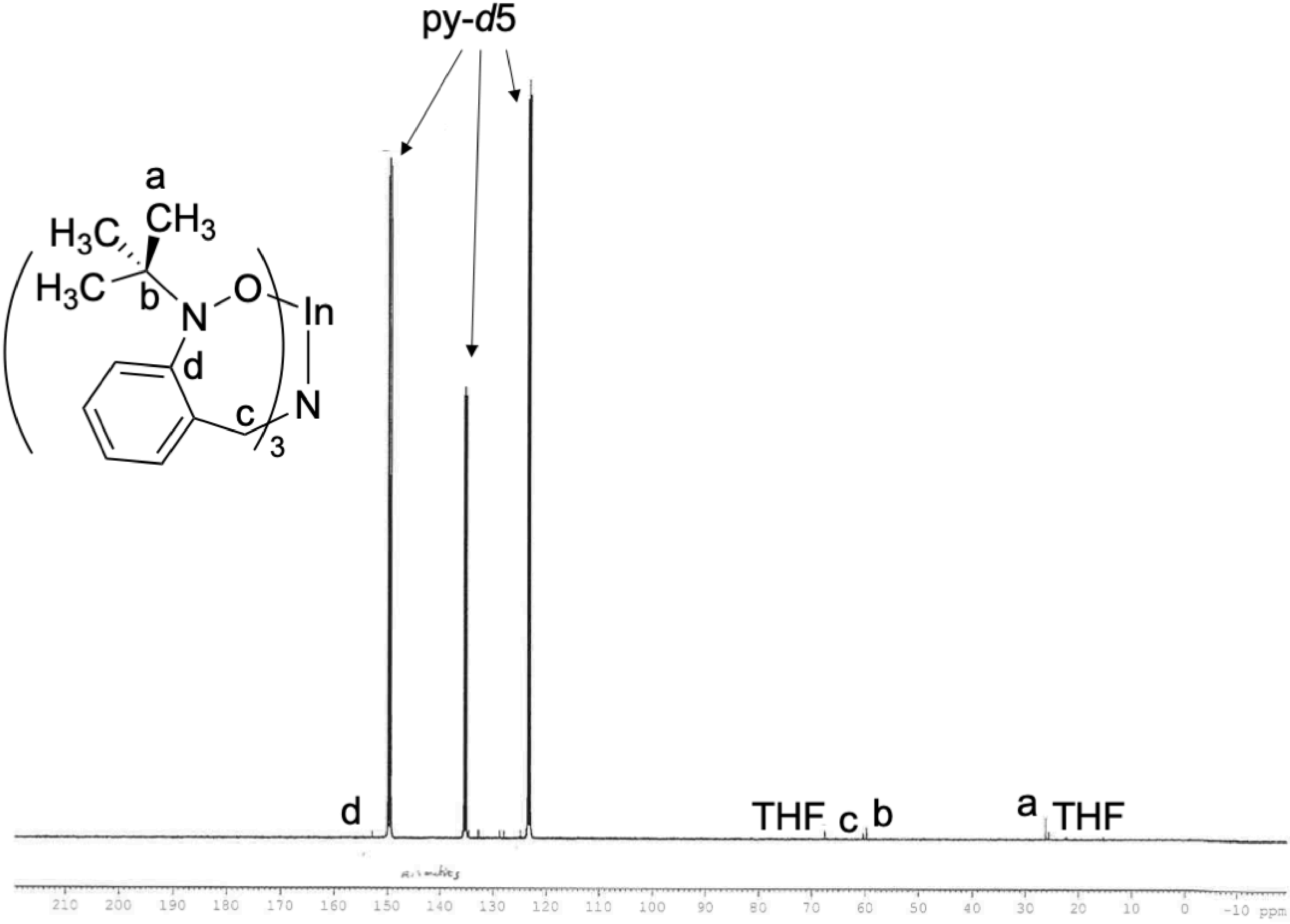

Figure S6. $^{13}C\{^{1}H\}$ NMR spectrum of $(TriNOx^{3-})In$ (**3**) in pyridine-*d*5.

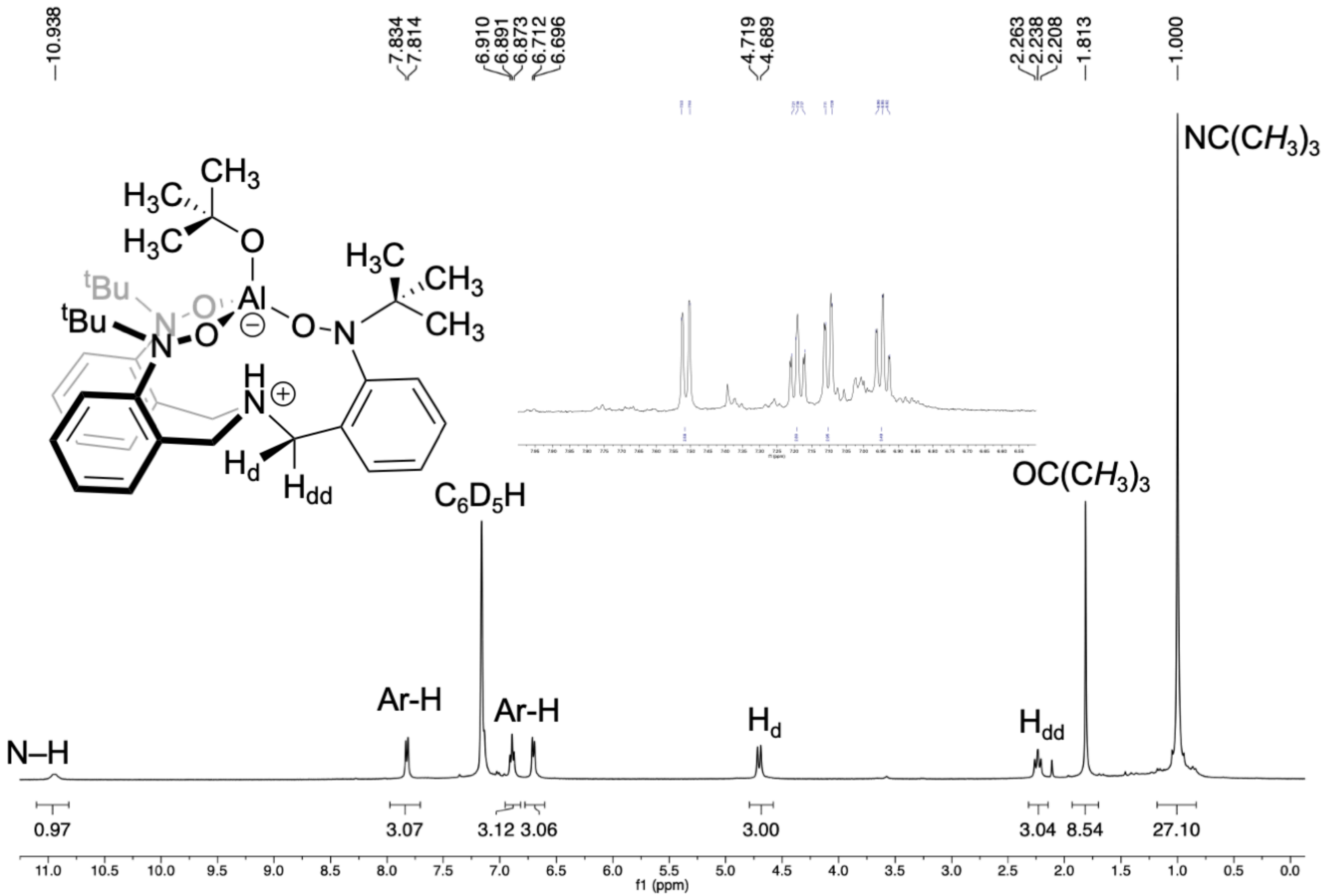

Figure S7. ^{1}H NMR spectrum of (HTriNOx^{2-})Al–O^{t}Bu (**4**) in C_6D_6. The insert shows the aromatic region of the ^{1}H NMR of **4** in THF-*d8*.

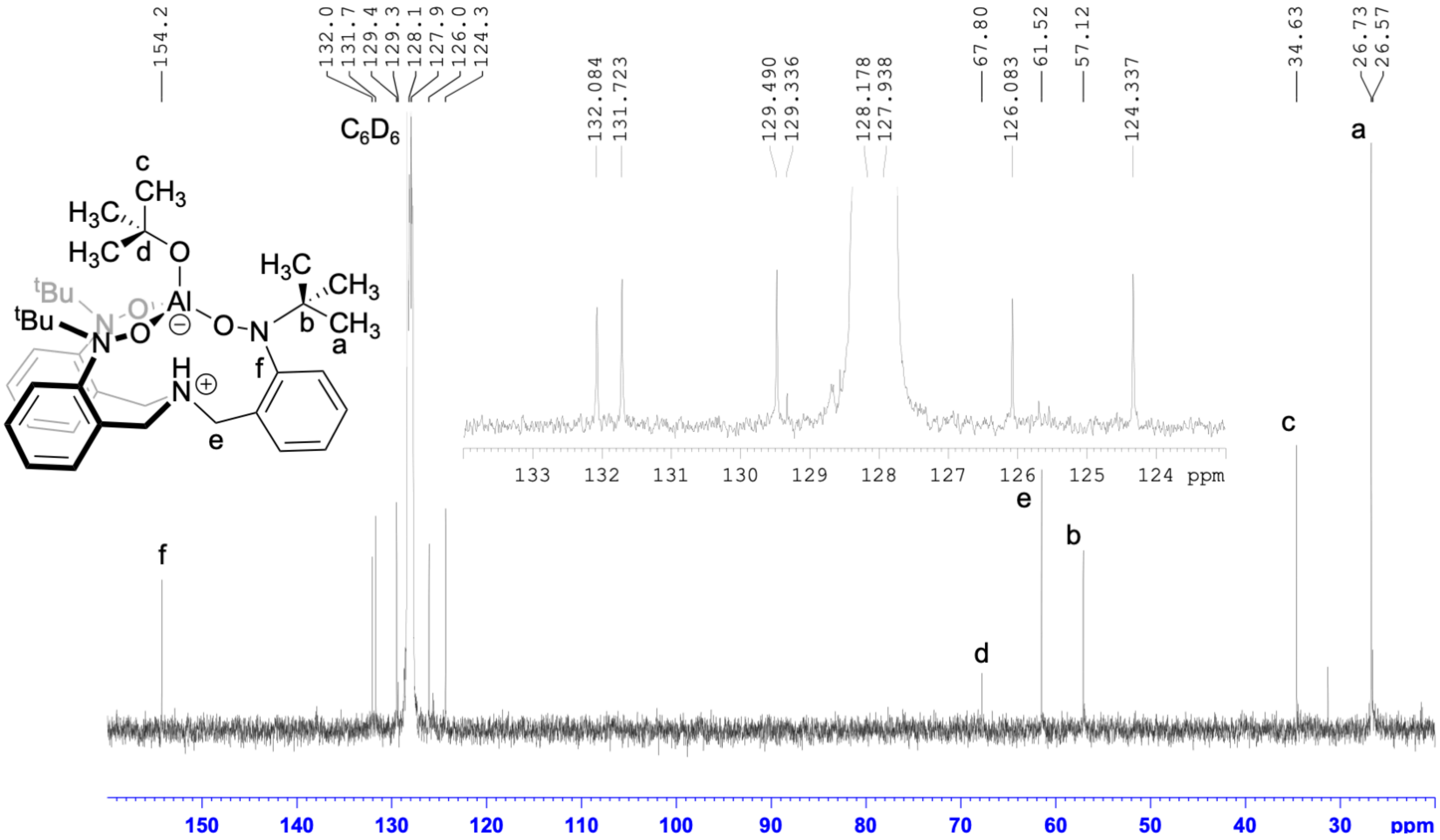

Figure S8. $^{13}C\{^{1}H\}$ NMR spectrum of (HTriNOx^{2-})Al–O^{t}Bu (**4**) in C_6D_6.

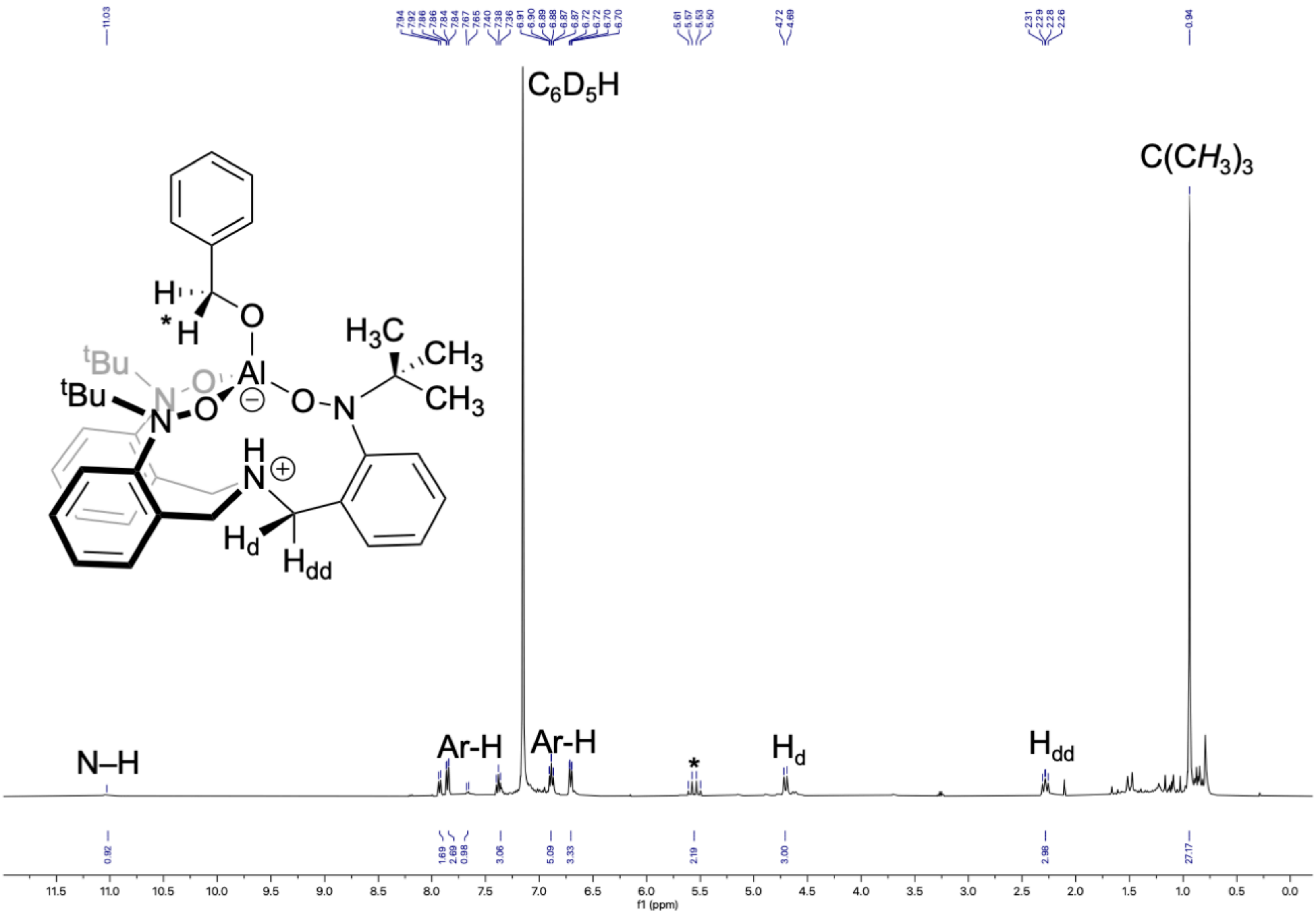

Figure S9. ^{1}H NMR spectrum of (HTriNOx^{2-})Al–OBn (**6**) in C_6D_6.

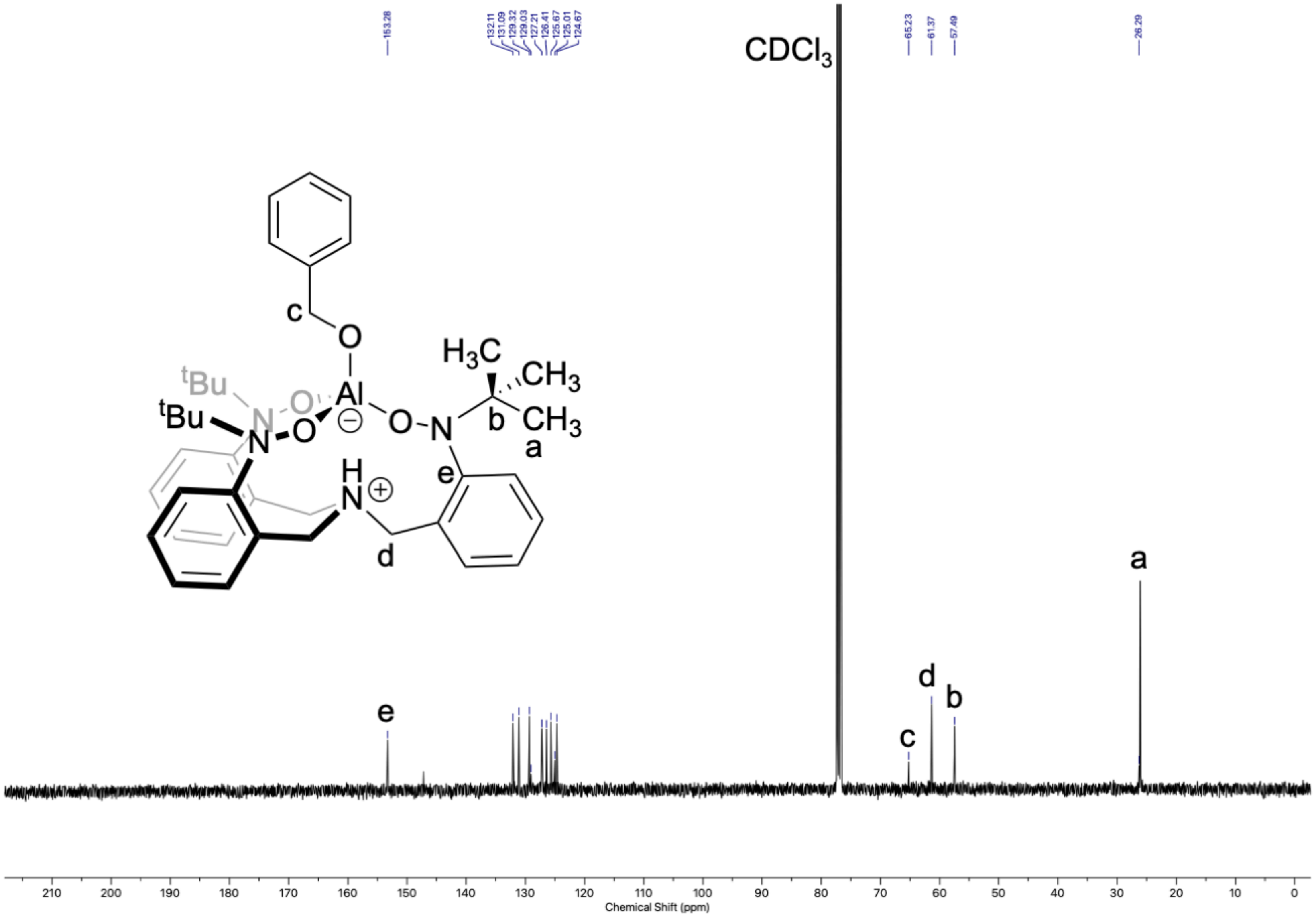

Figure S10. $^{13}C\{^{1}H\}$ NMR spectrum of (HTriNOx^{2-})Al–OBn (**6**) in $CDCl_3$.

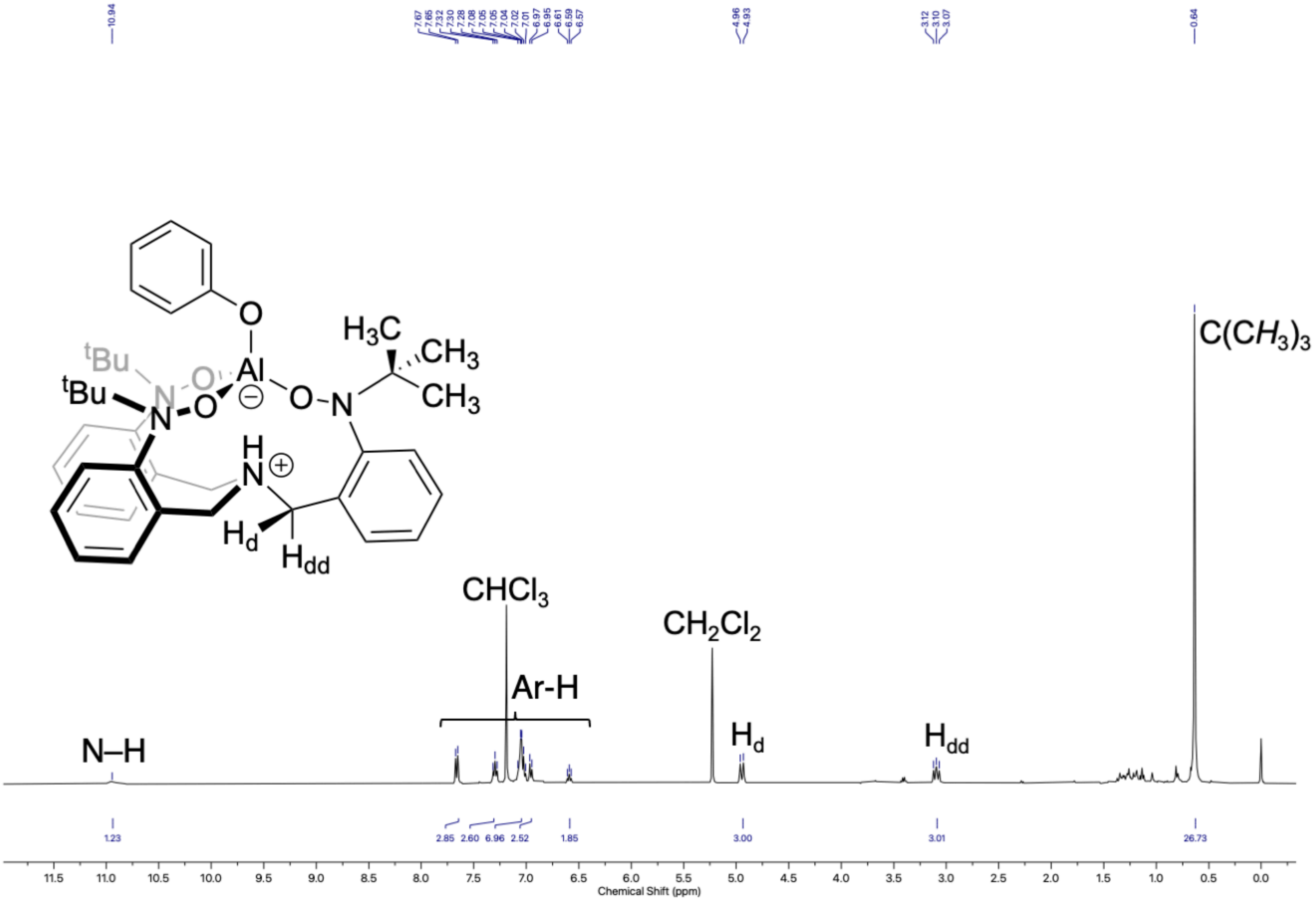

Figure S11. ^{1}H NMR spectrum of (HTriNOx^{2-})Al–OPh (**8**) in $CDCl_3$.

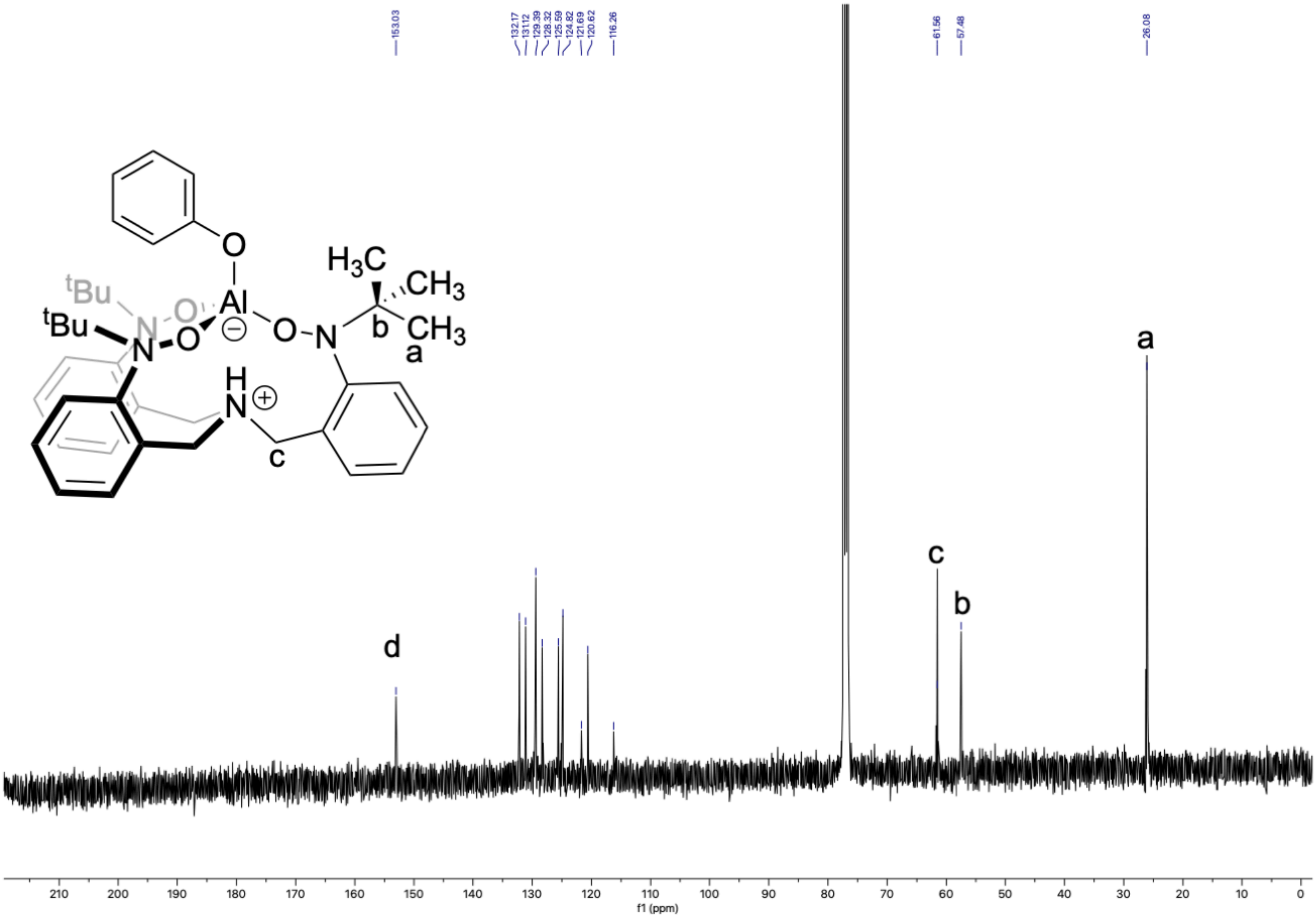

Figure S12. $^{13}C\{^{1}H\}$ NMR spectrum of $(HTriNOx^{2-})Al–OPh$ (**8**) in $CDCl_3$.

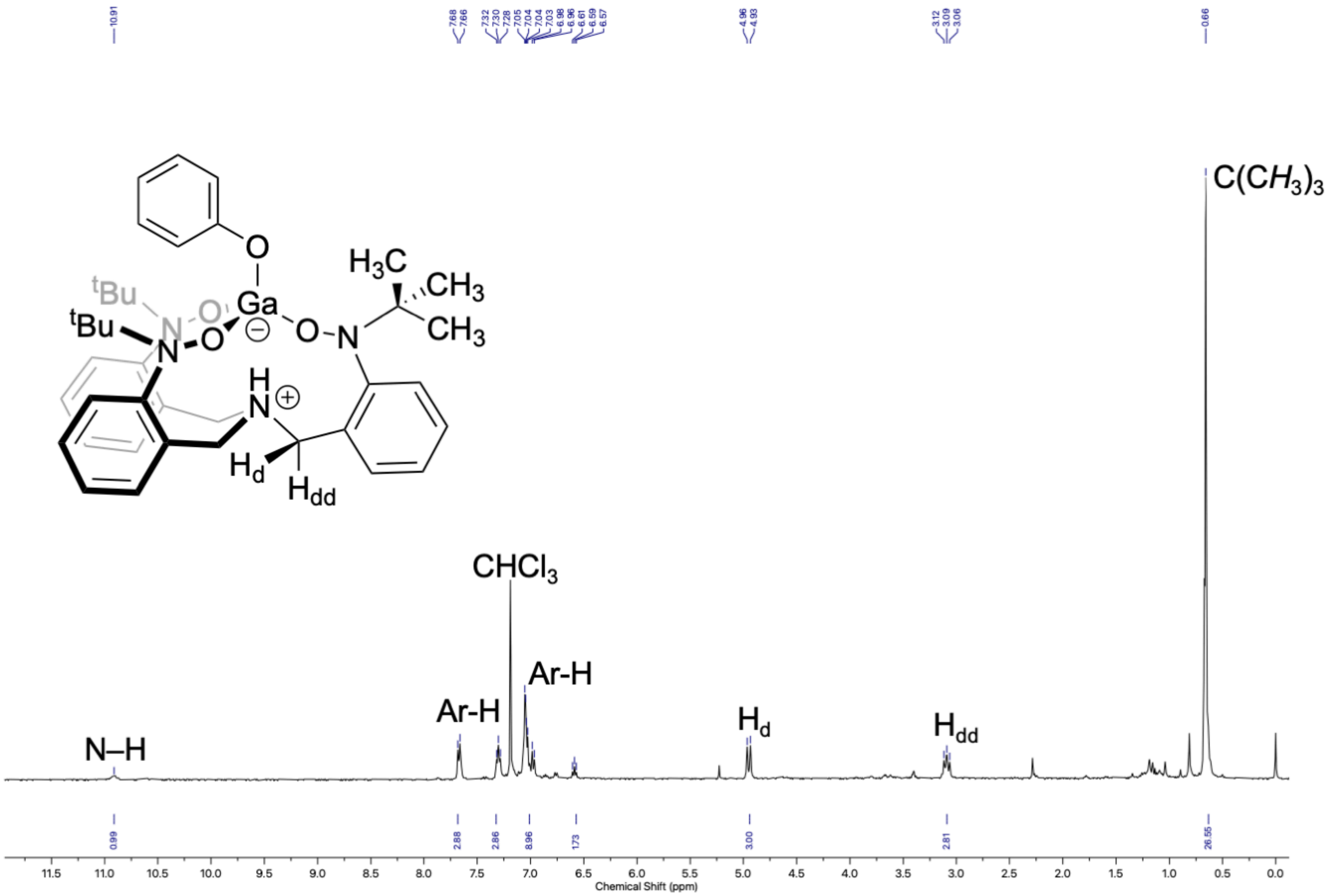

Figure S13. ^{1}H NMR spectrum of (HTriNOx^{2-})Ga–OPh (**9**) in $CDCl_3$.

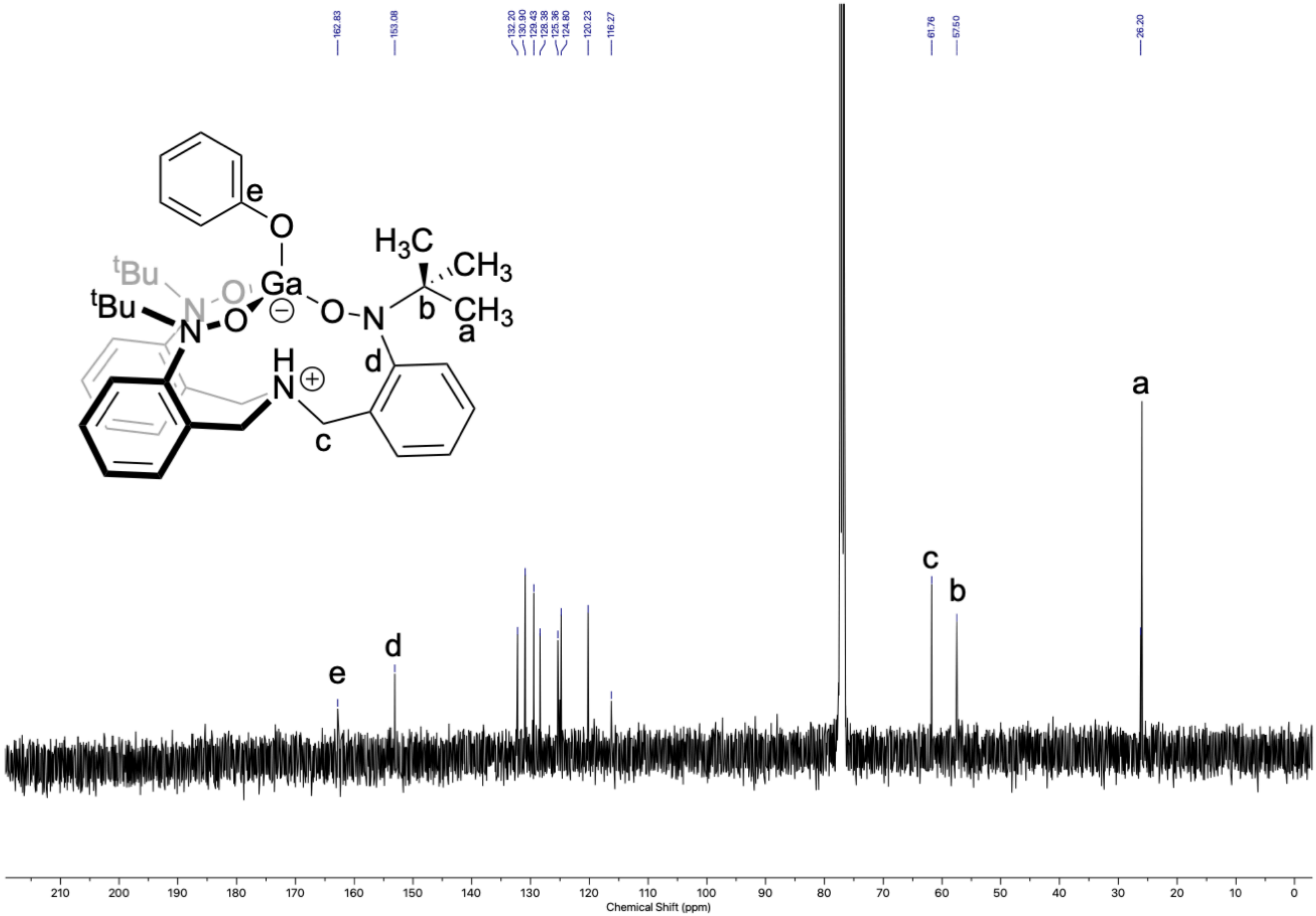

Figure S14. $^{13}C\{^1H\}$ NMR spectrum of (HTriNOx^{2-})Ga–OPh (**9**) in $CDCl_3$.

Protocol for determination of the Lewis acid acceptor numbers for 1 and 2: 0.25 mL of a 9 mM solution of either **1** or **2** (2.25×10^{-3} mmol) in C_6D_6 was added to a vial containing the given triethylphosphine chalcogenide (4.5×10^{-3} mmol) in 0.25 mL of C_6D_6. The reactions were stirred for 30 min and then analyzed by $^{31}P\{^1H\}$ NMR spectroscopy.

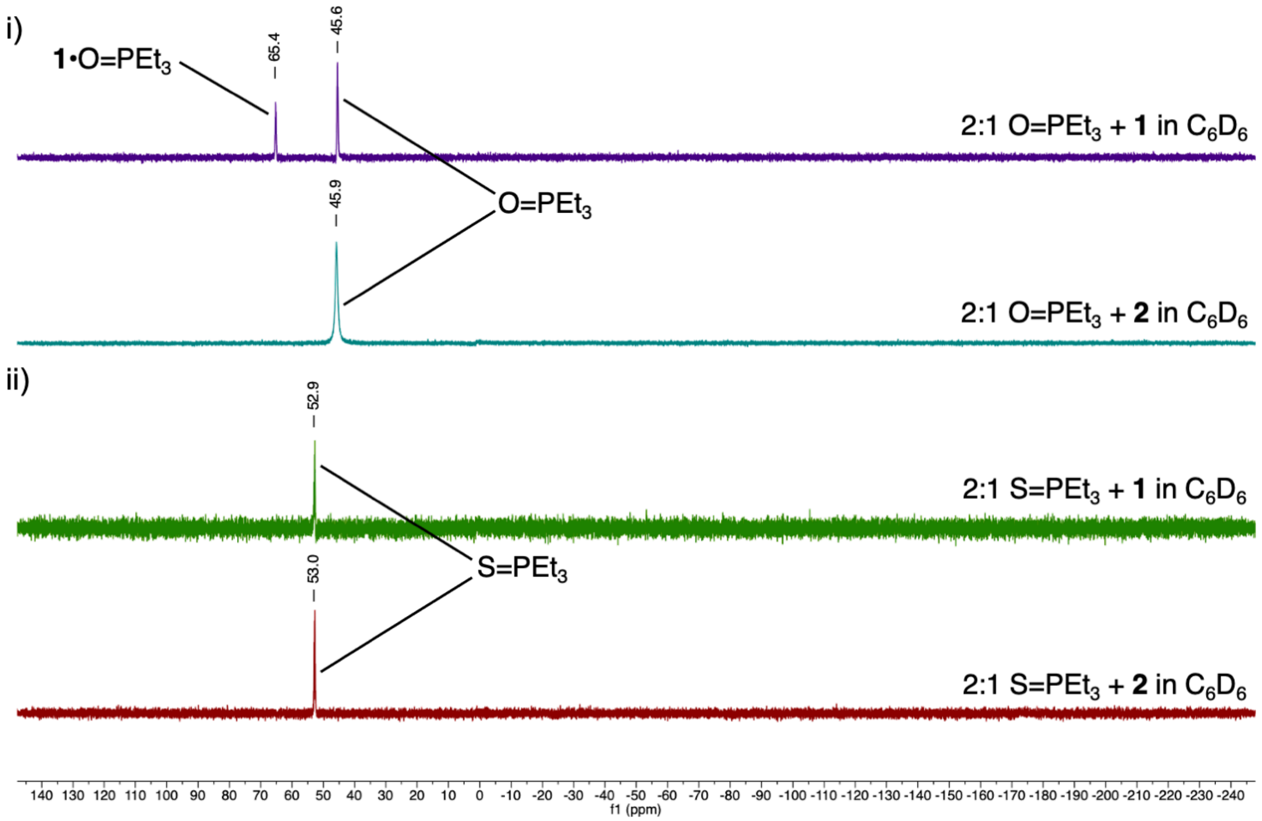

Figure S15. Stacked plot of the $^{31}P\{^1H\}$ NMR spectra of the mixtures of Et_3PO (i) and Et_3PS (ii) experiments run to evaluate the Lewis acidity of complexes **1** and **2**.

Protocol for the VT-NMR studies of Complexes 4, 6, and 8: ~5 mg of complex was dissolved in ~0.75 mL of NMR solvent (C_6D_6 for **4**; $CDCl_3$ for **6** and **8**). The solution was transferred into an NMR tube equipped with a Teflon J. Young valve and the sample was allowed to equilibrate at 293 K and a 1H NMR spectrum was collected. Then, ^{1}H NMR spectra were recorded at 303K, 313K, 323K, 333K, 343K, and 353K; before each collection, the sample was allowed to thermally equilibrate for 30 min and then the sample was shimmed prior to collection of the spectra.

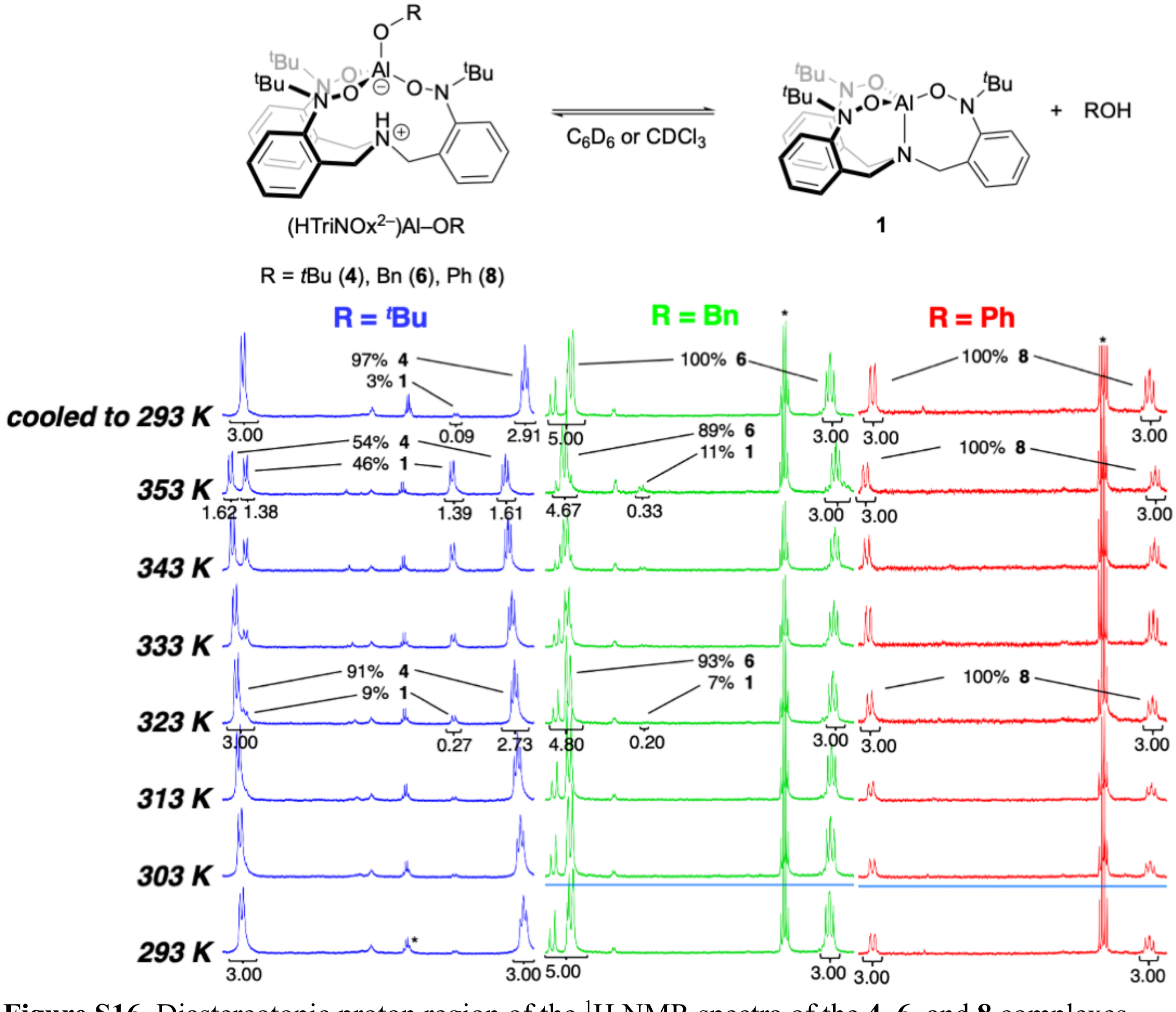

Figure S16. Diastereotopic proton region of the ^{1}H NMR spectra of the **4, 6**, and **8** complexes over the temperature range 293–353 K. The samples all contain diethyl ether (marked with an *) that remains throughout the experiment.

Protocol for the van't Hoff experiment of 1 + ^{t}BuOH: Into a vial was added 500 μL of a 9 mM stock solution of **1** in C_6D_6 along with 125 μL of a 6 mM stock solution of hexamethylcyclotrisiloxane as an internal standard, also in C_6D_6. Then, 125 μL of a 36 mM solution of *tert*-butanol in C_6D_6 was added and the reaction was allowed to stir at room temperature for 24 h. The reaction mixture was then transferred to a J-young capped NMR tube and loaded into the NMR spectrometer. ^{1}H NMR spectra were collected at each indicated temperature. In all cases, the reaction was allowed to thermally equilibrate at the given temperature for 30 min prior to collection of the spectra. The concentration of the metal complexes **1** and **4** were determined by comparison of the integrations of the resonances for the *t*Bu and diasteriotopic CH_2 protons of the TriNOx ligands in each complex relative to internal standard. K_{eq} values were calculated according to the formula:

$$K_{eq} = \frac{[4]}{[1][tBuOH]} \qquad \text{(Eq. S1)}$$

Protocol for the determining the K_{eq} values for the reactions of 2 with alcohols. For each experiment, 500 μL of a 9 mM stock solution of **2** in $CDCl_3$ was dispensed into a vial along with 125 μL of a 6 mM stock solution of hexamethylcyclotrisiloxane as an internal standard also in $CDCl_3$. Then, 125 μL of a 36 mM stock solution of the appropriate alcohol in $CDCl_3$ was dispensed into the vial. The reaction was allowed to stir at room temperature for 24 hours after which the reaction was transferred to an NMR tube and analyzed by ^{1}H NMR spectroscopy. K_{eq} values were calculated according to the formula:

$$K_{eq} = \frac{[(HTriNOx)MOR]}{[2][ROH]} \qquad \text{(Eq. S2)}$$

The concentration of the metal complexes were determined by comparison of the integrations of the ligand NMR signatures to internal standard. For **2** the concentration was taken as the average value determined from comparison to the *t*Bu groups as well as both diastereotopic protons of the bridgehead CH_2 groups. The concentration of the $(HTriNOx^{2-})$Ga–OR complexes were similarly determined by the average of the value found for the *t*Bu groups as well as both diastereotopic protons of the bridgehead CH_2 groups, as well as with any easily identifiable NMR handles in the R group of the resultant alkoxide ligand. The concentration of unreacted alcohol was assumed to be equal to that unreacted complex **2**.

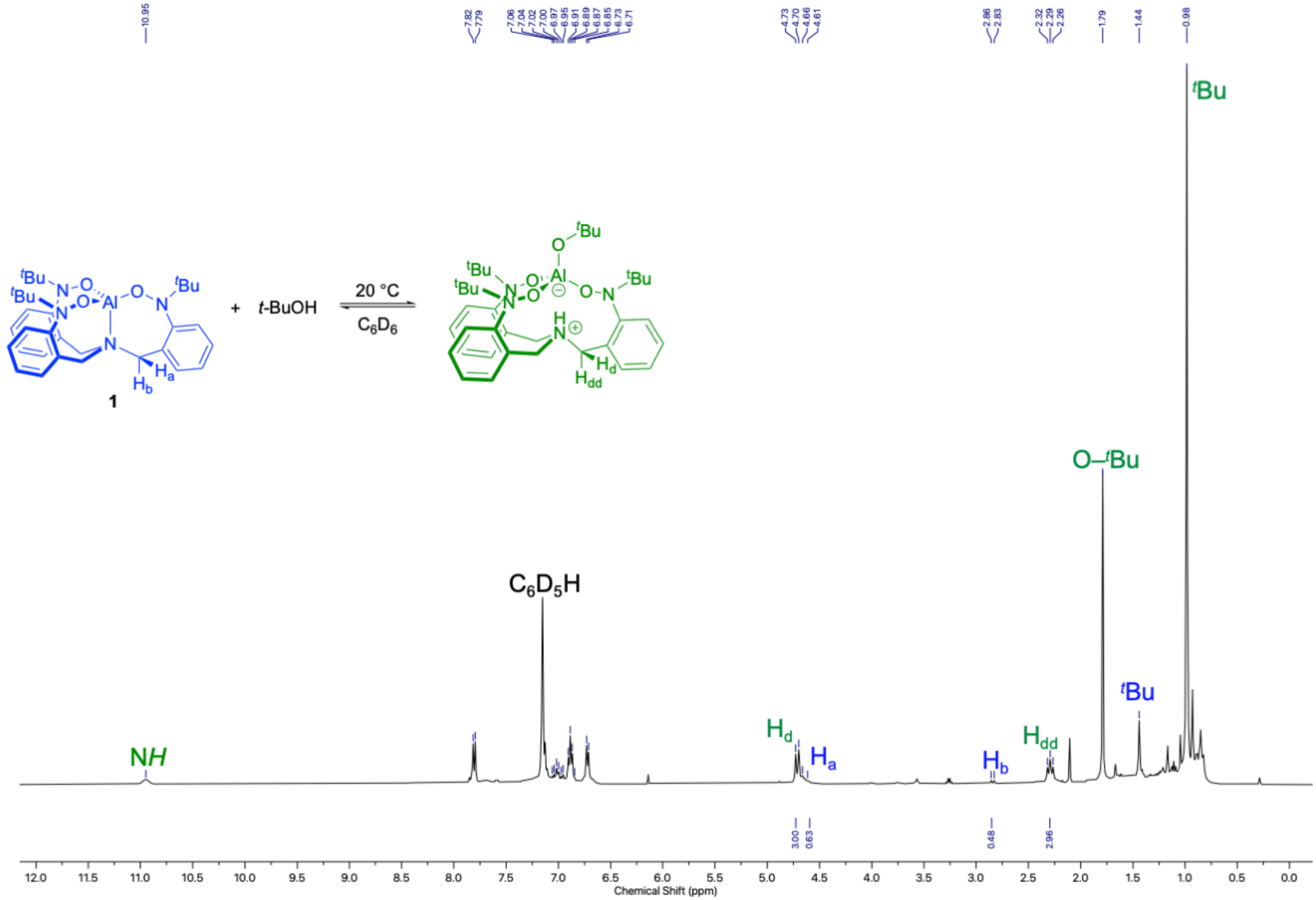

Figure S17. ^{1}H NMR spectrum of a 1:1 mixture of **1:***t*-BuOH. Taken in C_6D_6 and recorded after 24 hours of stirring at room temperature.

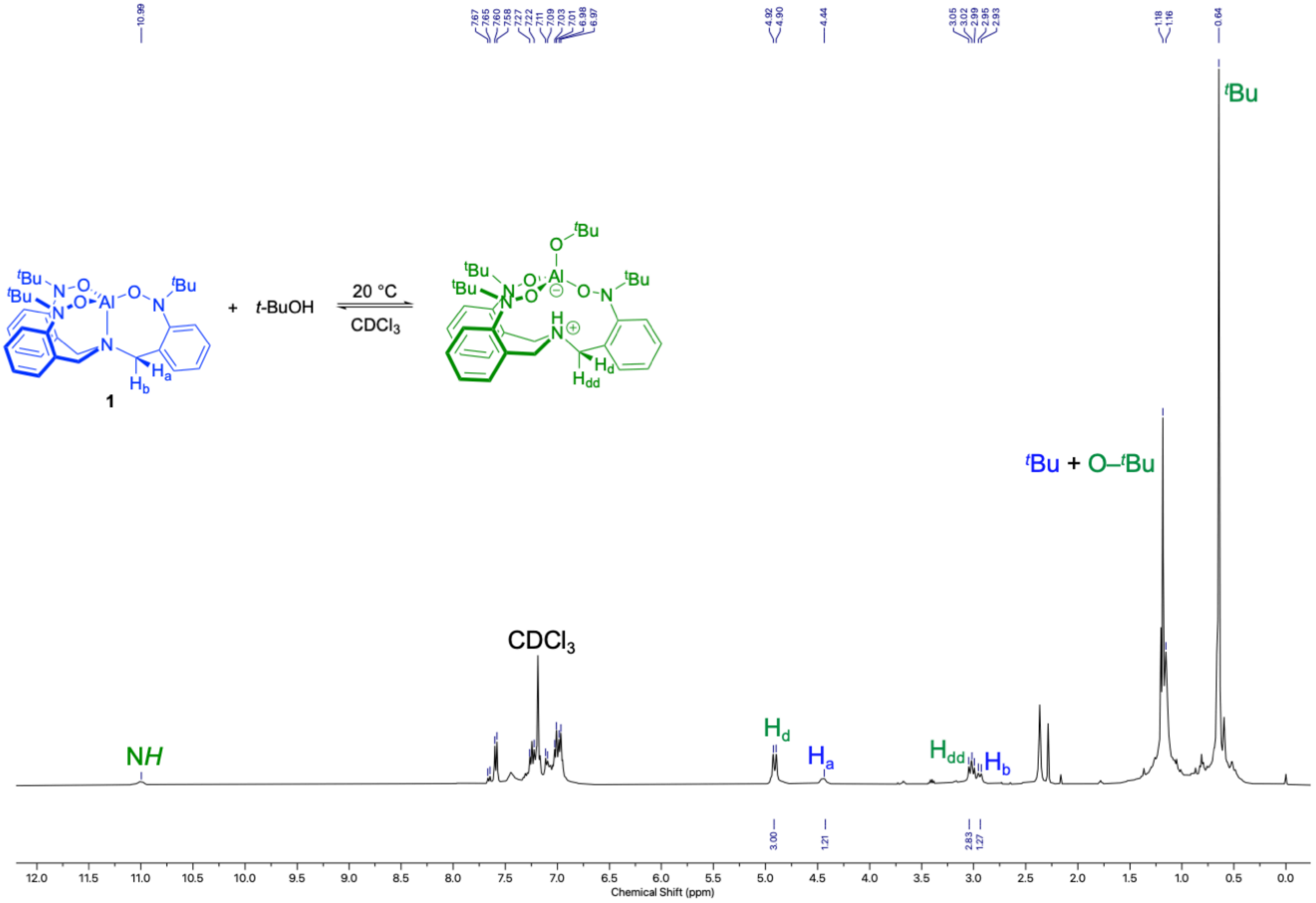

Figure S18. ^{1}H NMR spectrum of a 1:1 mixture of **1:***t*-BuOH. Taken in $CDCl_3$ and recorded after 24 hours of stirring at room temperature.

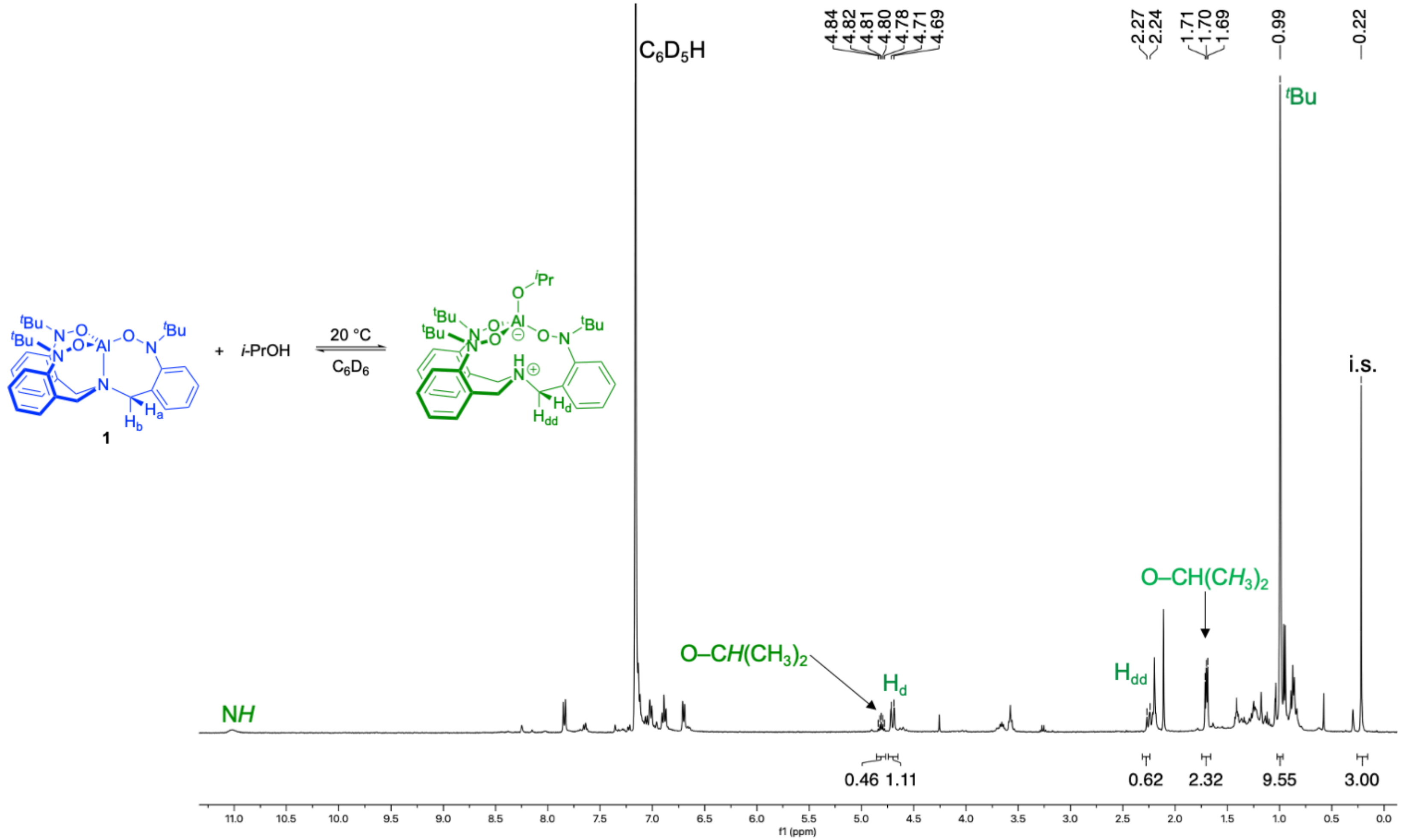

Figure S19. ^{1}H NMR spectrum of a 1:1 mixture of **1:***i*-PrOH. Taken in C_6D_6 and recorded after 24 hours of stirring at room temperature.

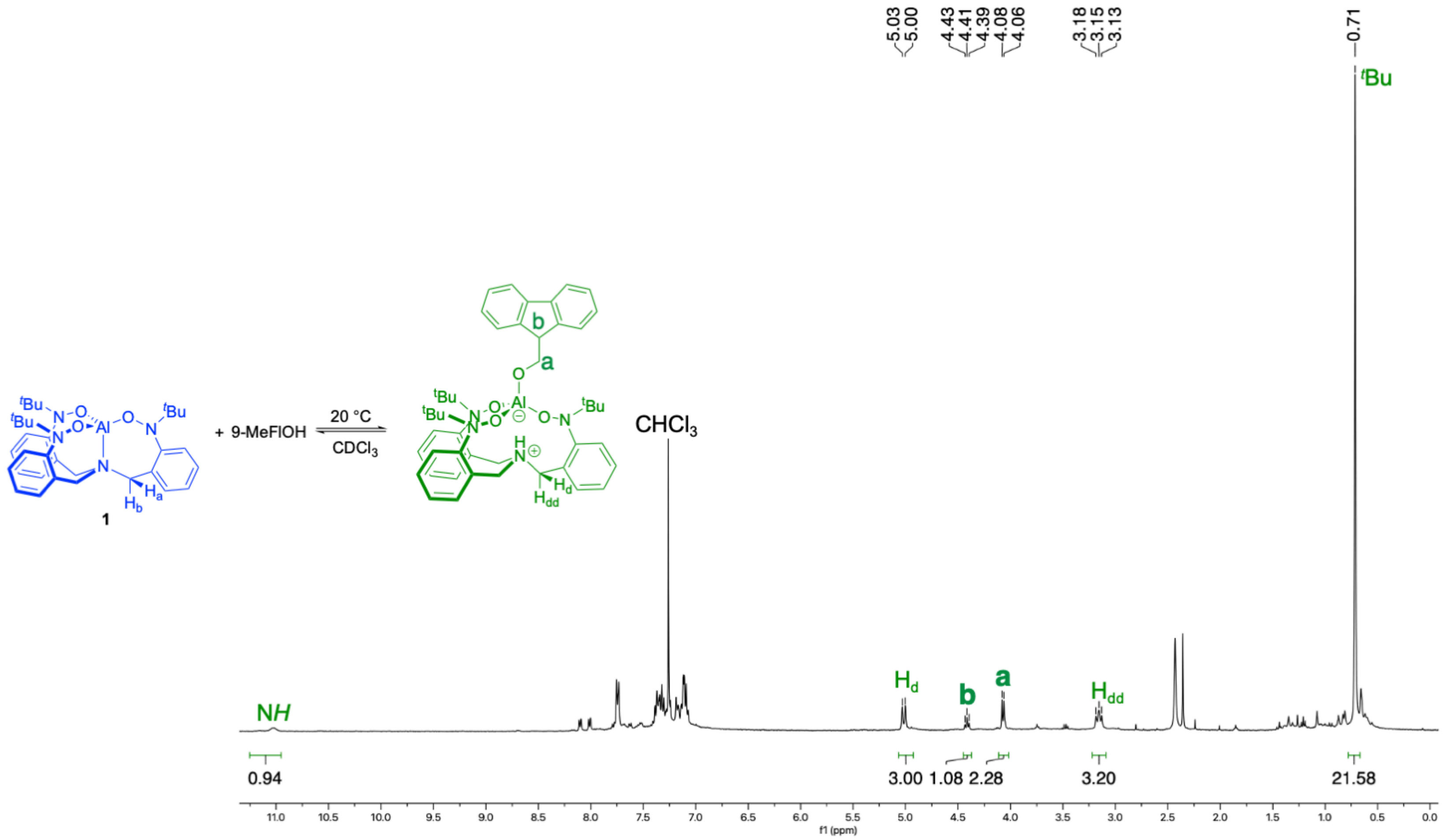

Figure S20. ^{1}H NMR spectrum of a 1:1 mixture of **1**:9-fluorenemethanol. Taken in $CDCl_3$ and recorded after 24 hours of stirring at room temperature.

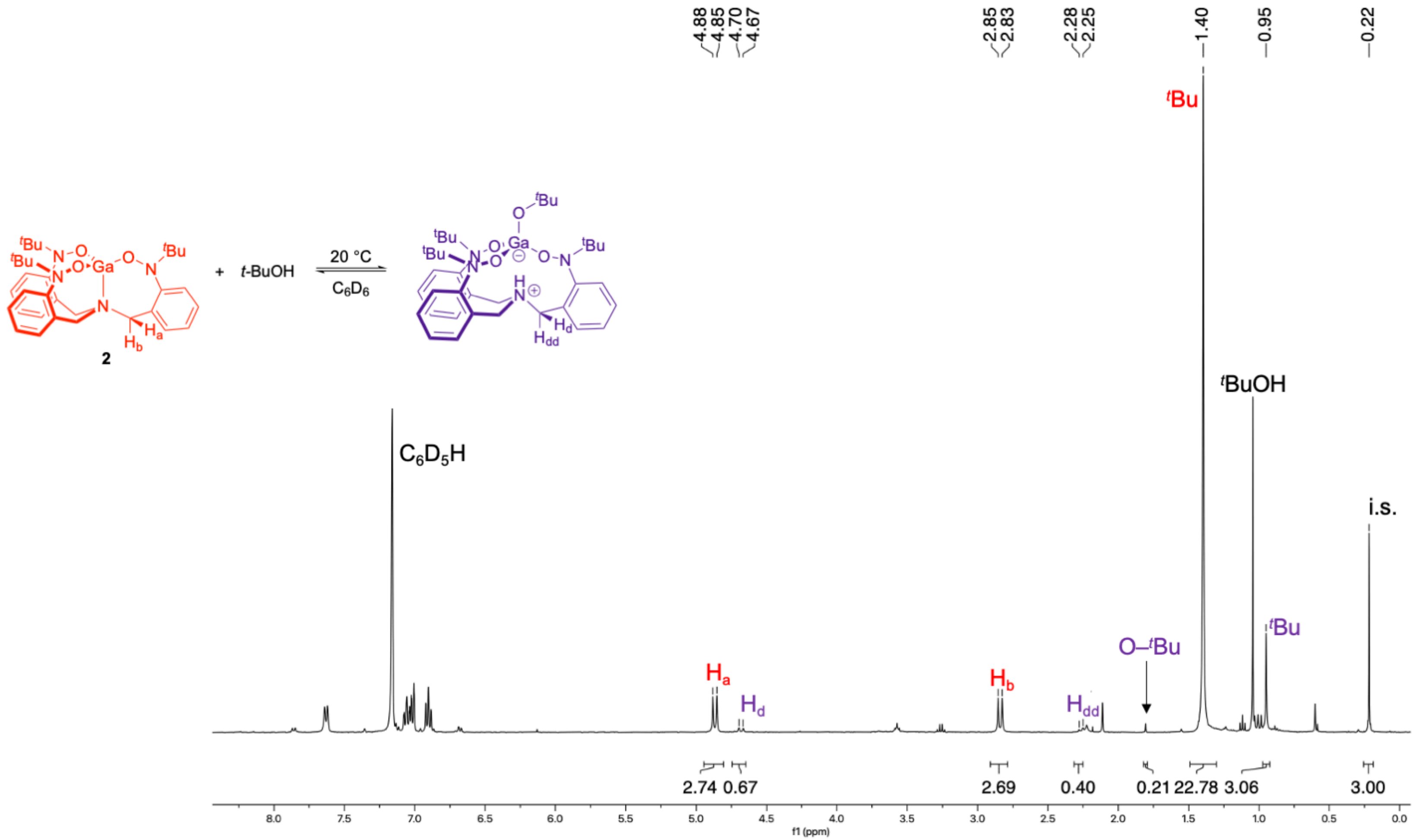

Figure S21. ^{1}H NMR spectrum of a 1:1 mixture of **2**:*t*-BuOH. Taken in C_6D_6 and recorded after 24 hours of stirring at room temperature (Table 1, entry 1).

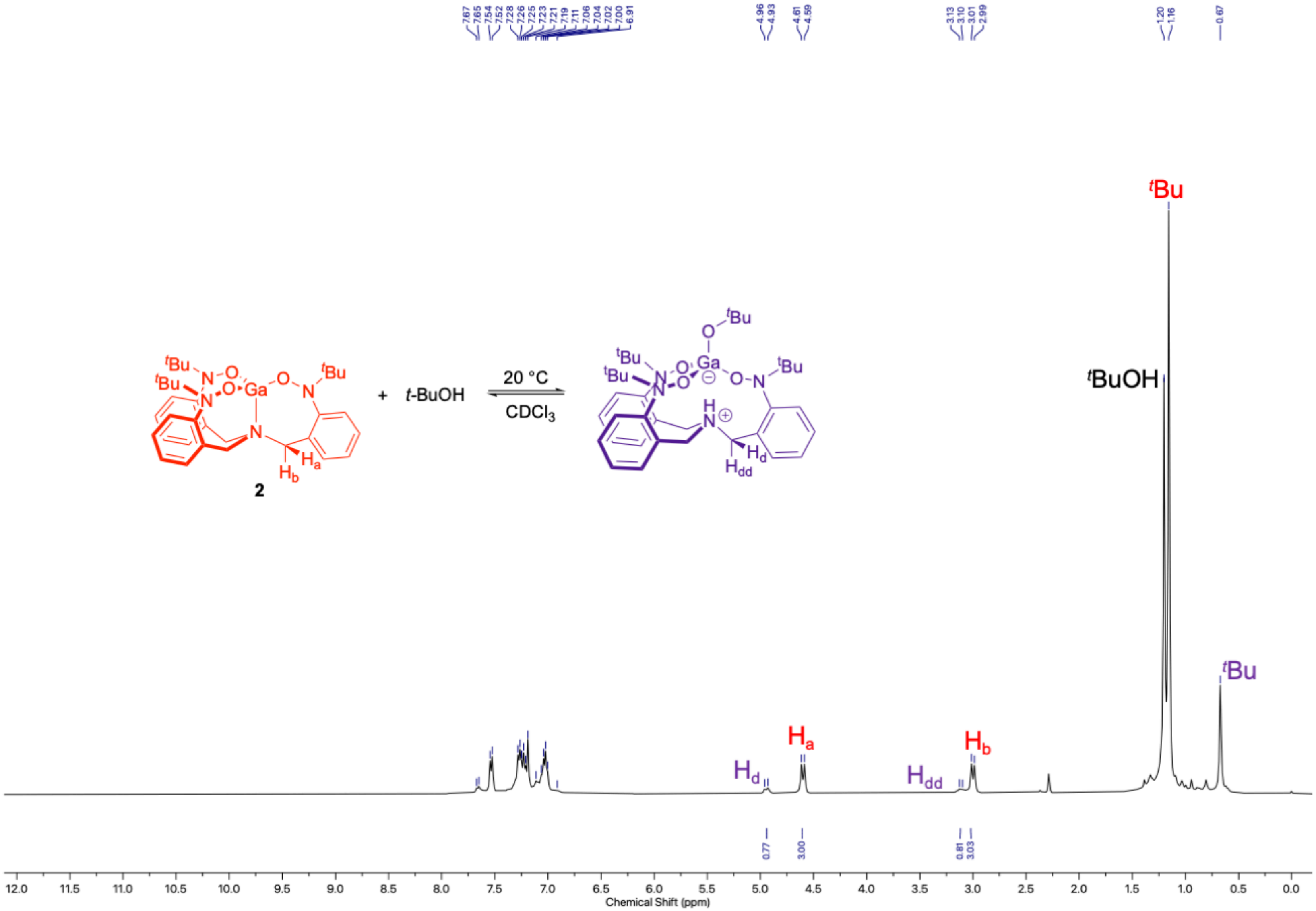

Figure S22. ^{1}H NMR spectrum of a 1:1 mixture of **2**:*t*-BuOH. Taken in $CDCl_3$ and recorded after 24 hours of stirring at room temperature.

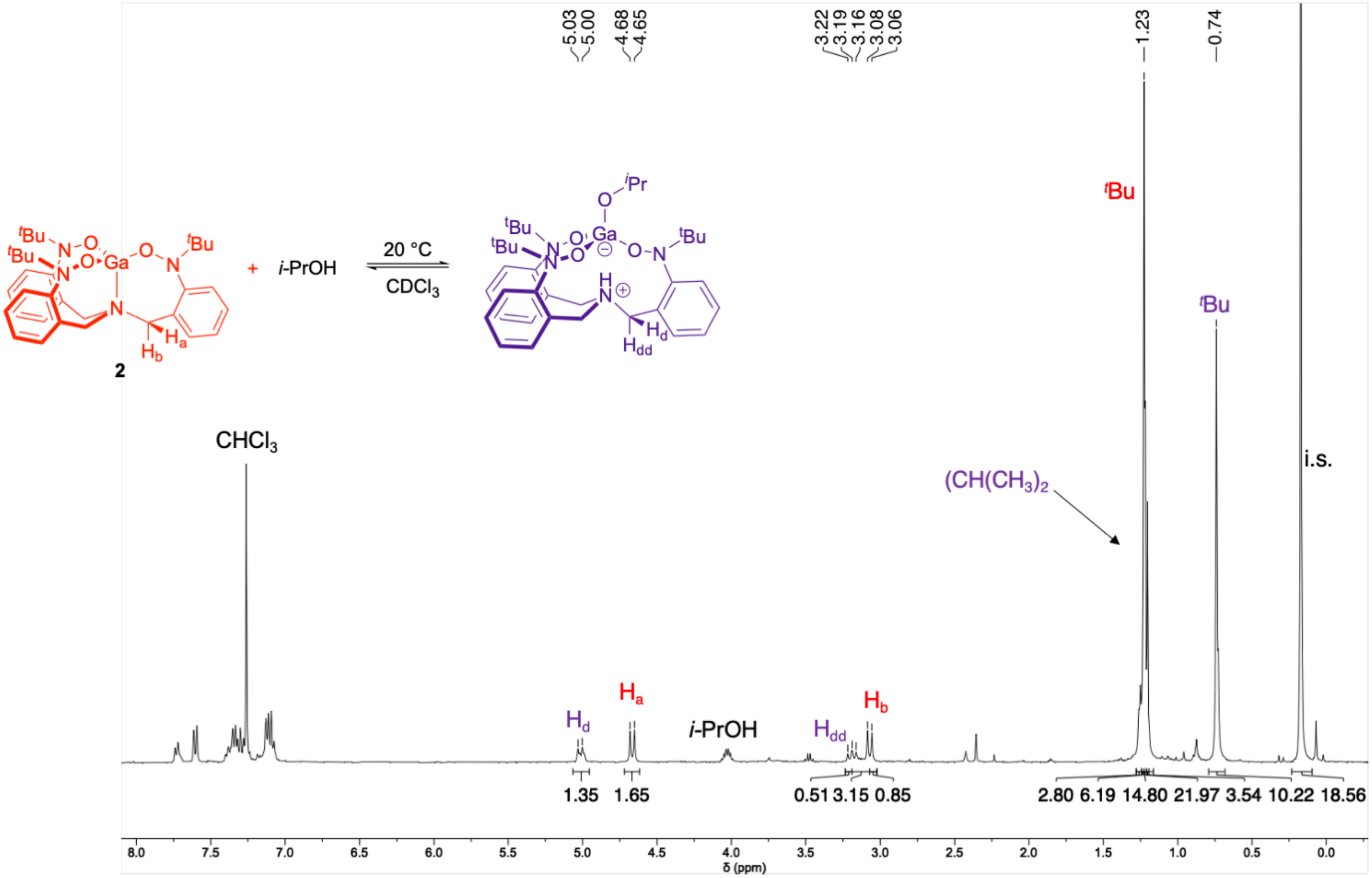

Figure S23. ^{1}H NMR spectrum of a 1:1 mixture of **2**:*i*-PrOH. Taken in $CDCl_3$ and recorded after 24 hours of stirring at room temperature (Table 1, entry 2).

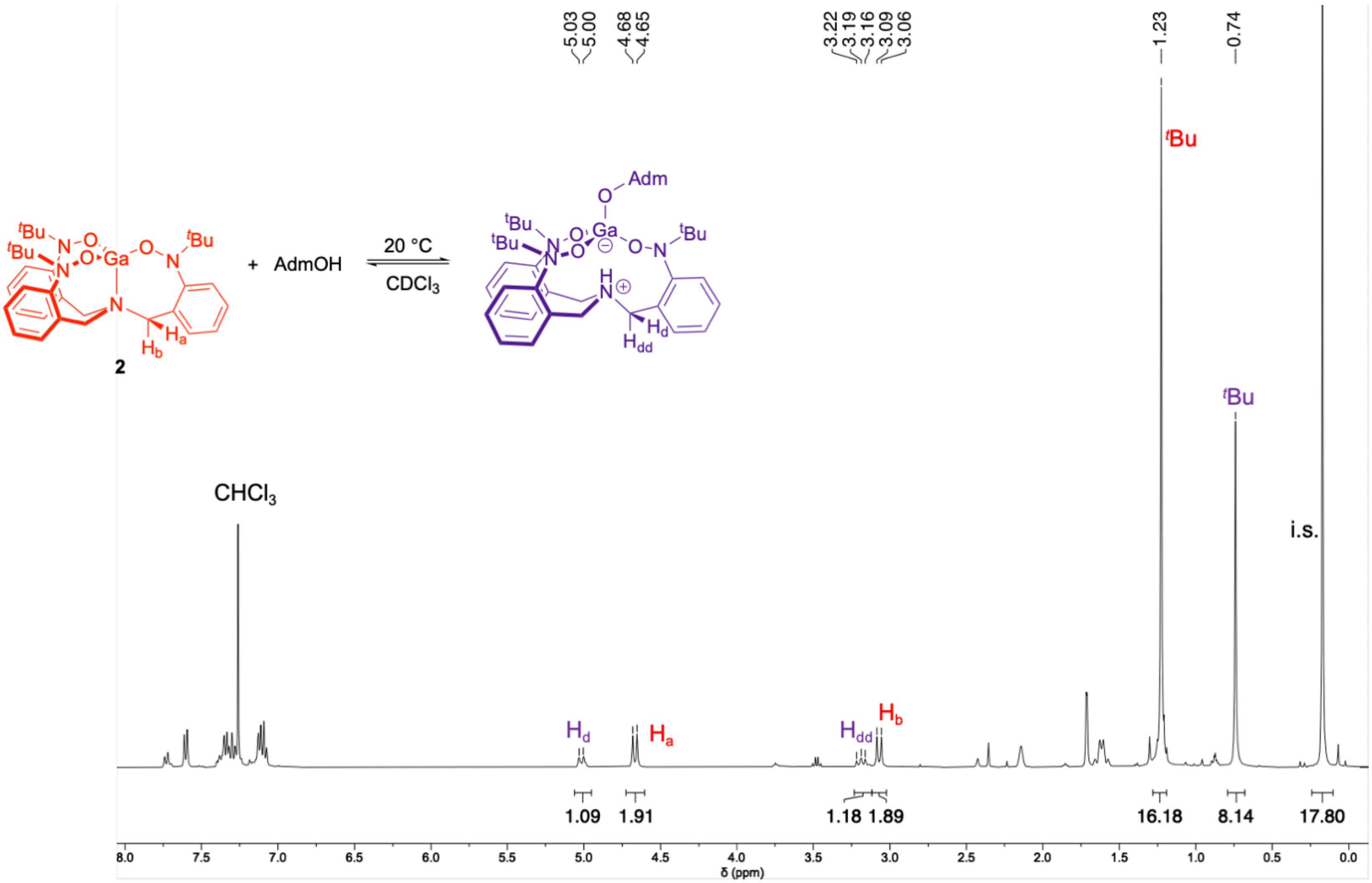

Figure S24. ^{1}H NMR spectrum of a 1:1 mixture of **2**:1-adamantanol. Taken in $CDCl_3$ and recorded after 24 hours of stirring at room temperature (Table 1, entry 3).

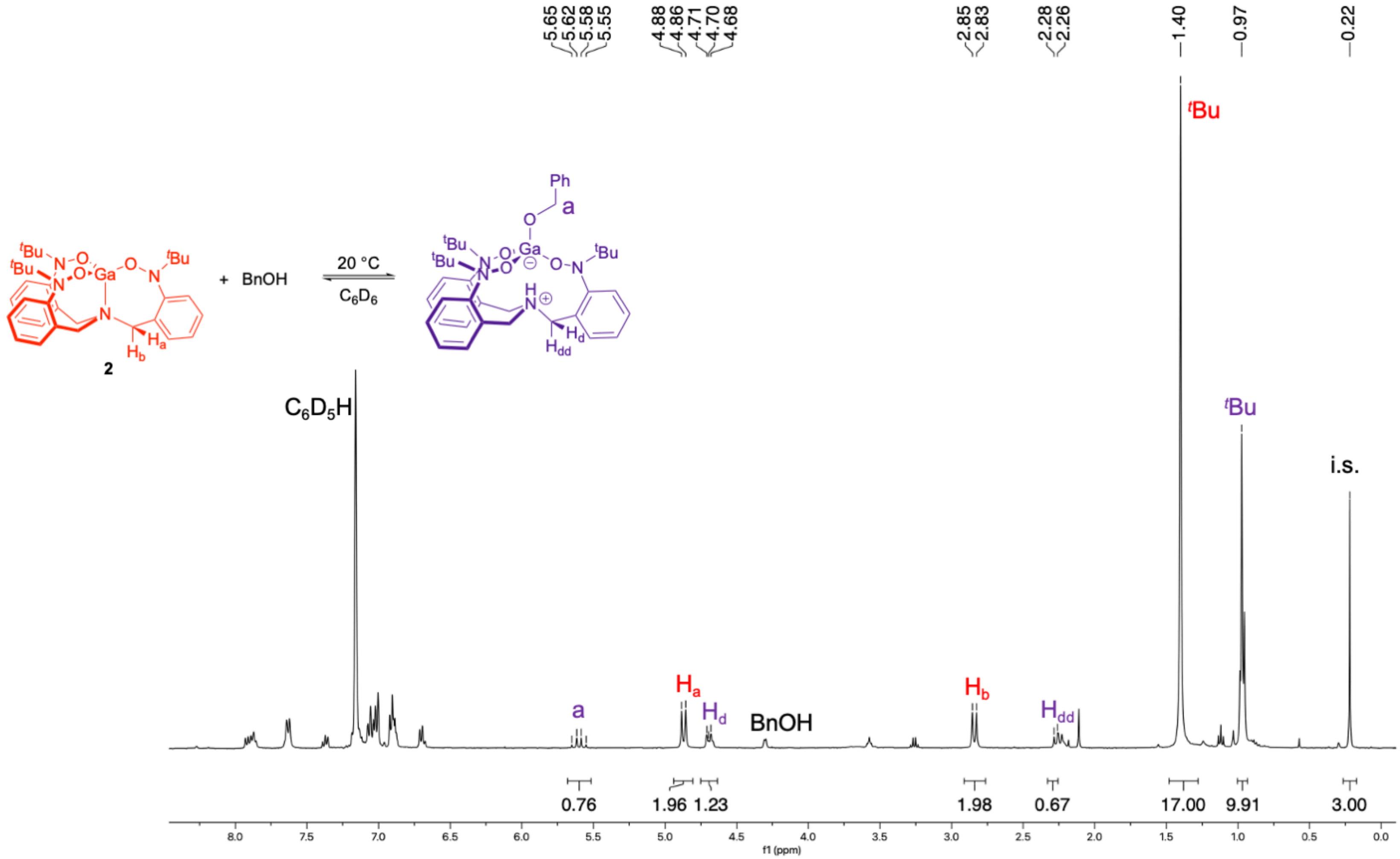

Figure S25. ¹H NMR spectrum of a 1:1 mixture of **2**:BnOH. Taken in C_6D_6 and recorded after 24 hours of stirring at room temperature (Table 1, entry 4).

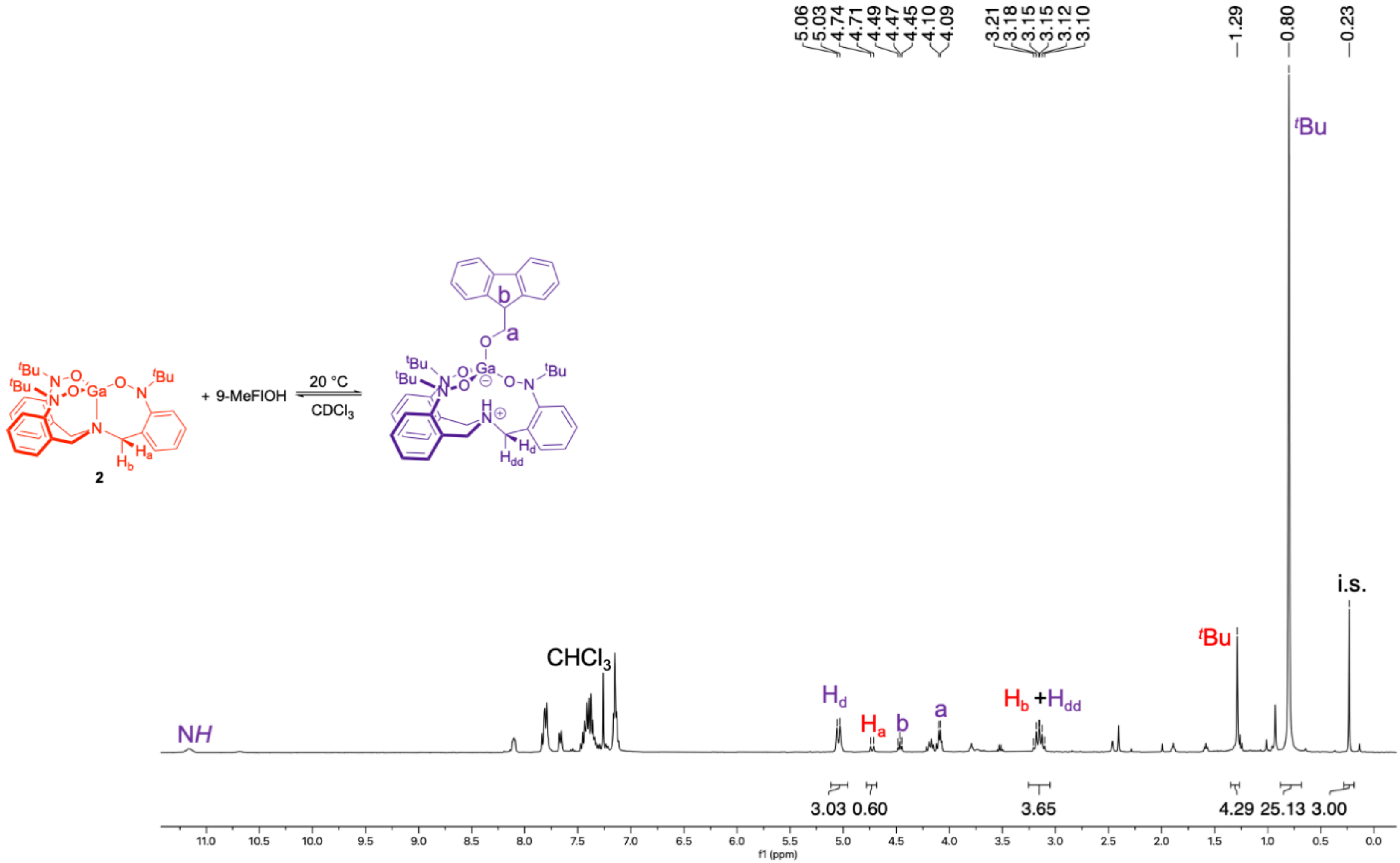

Figure S26. ^{1}H NMR spectrum of a 1:1 mixture of **2**:9-fluorenemethanol. Taken in $CDCl_3$ and recorded after 24 hours of stirring at room temperature (Table 1, entry 5).

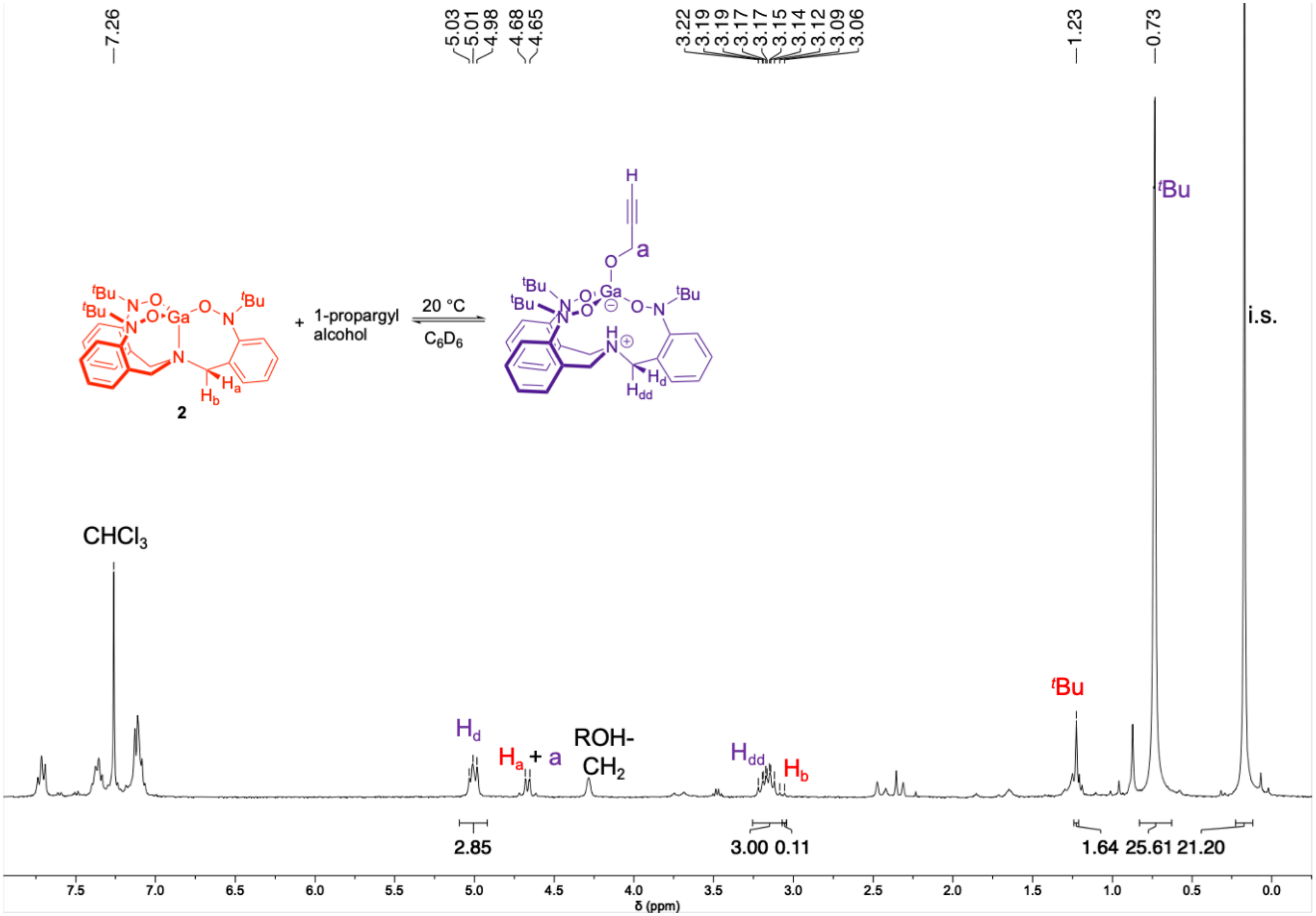

Figure S27. ^{1}H NMR spectrum of a 1:1 mixture of **2**:propargyl alcohol. Taken in $CDCl_3$ and recorded after 24 hours of stirring at room temperature (Table 1, entry 6).

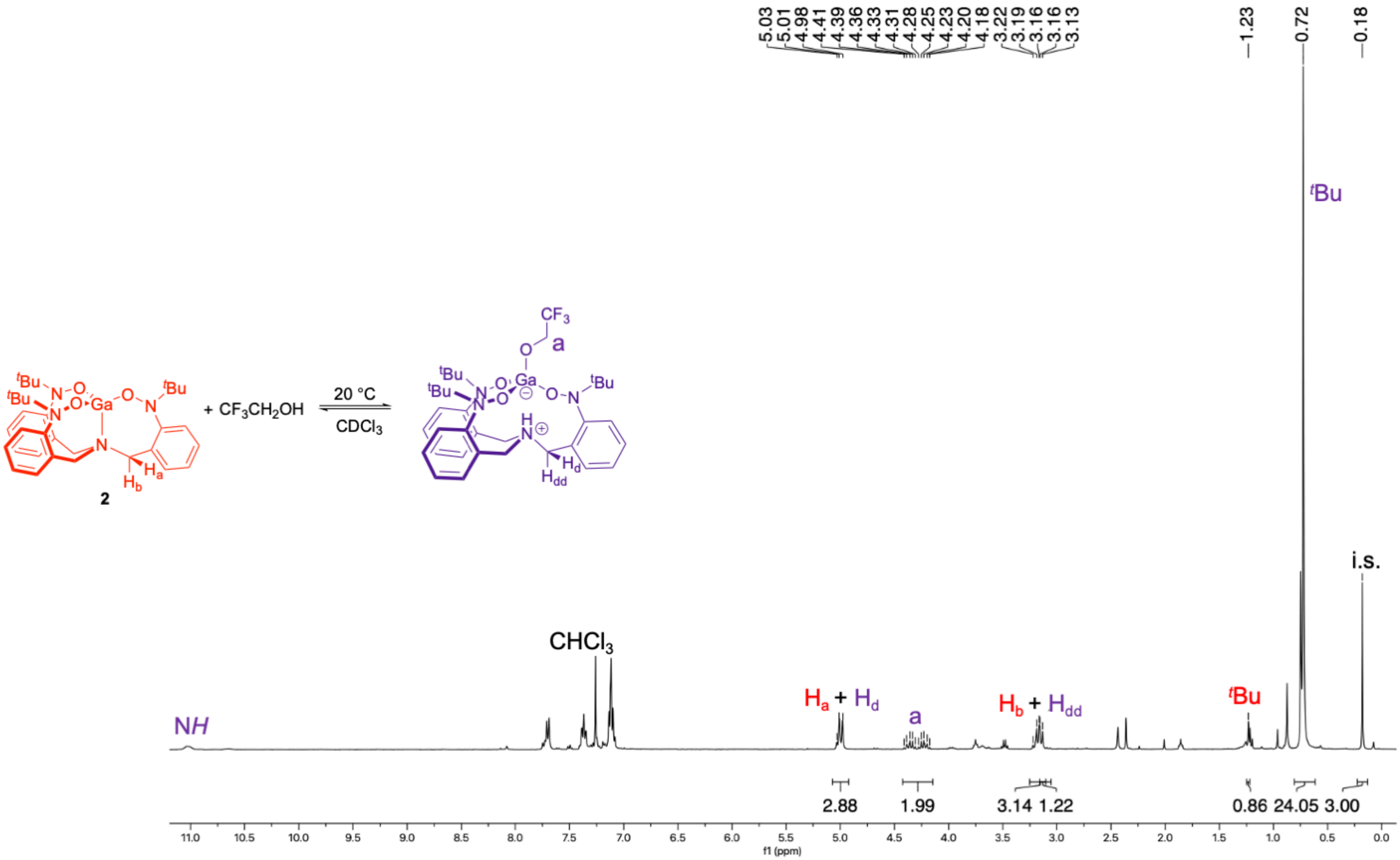

Figure S28. ^{1}H NMR spectrum of a 1:1 mixture of **2**:CF_3CH_2OH. Taken in $CDCl_3$ and recorded after 24 hours of stirring at room temperature (Table 1, entry 7).

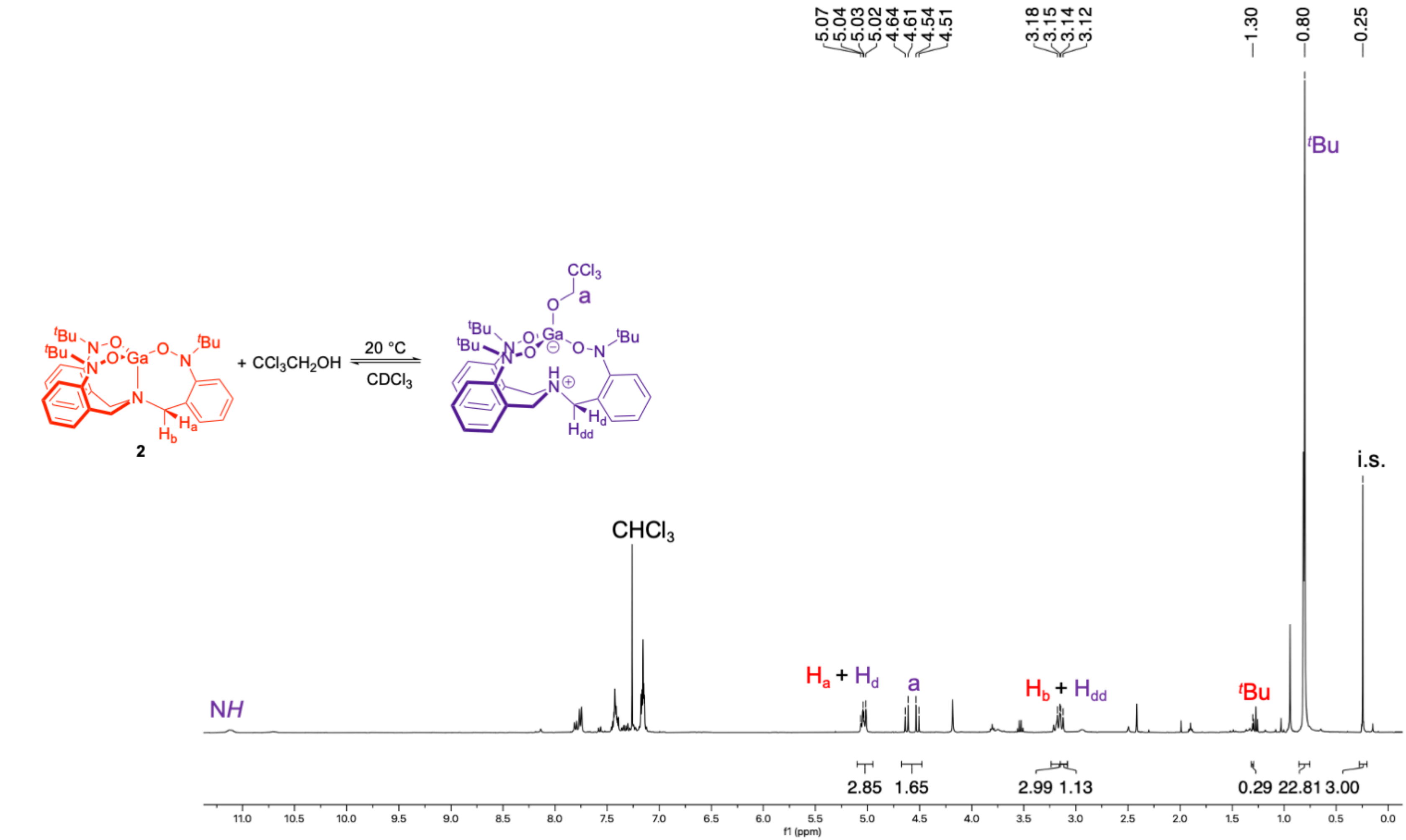

Figure S29. ^{1}H NMR spectrum of a 1:1 mixture of **2**:CCl_3CH_2OH. Taken in $CDCl_3$ and recorded after 24 hours of stirring at room temperature (Table 1, entry 8).

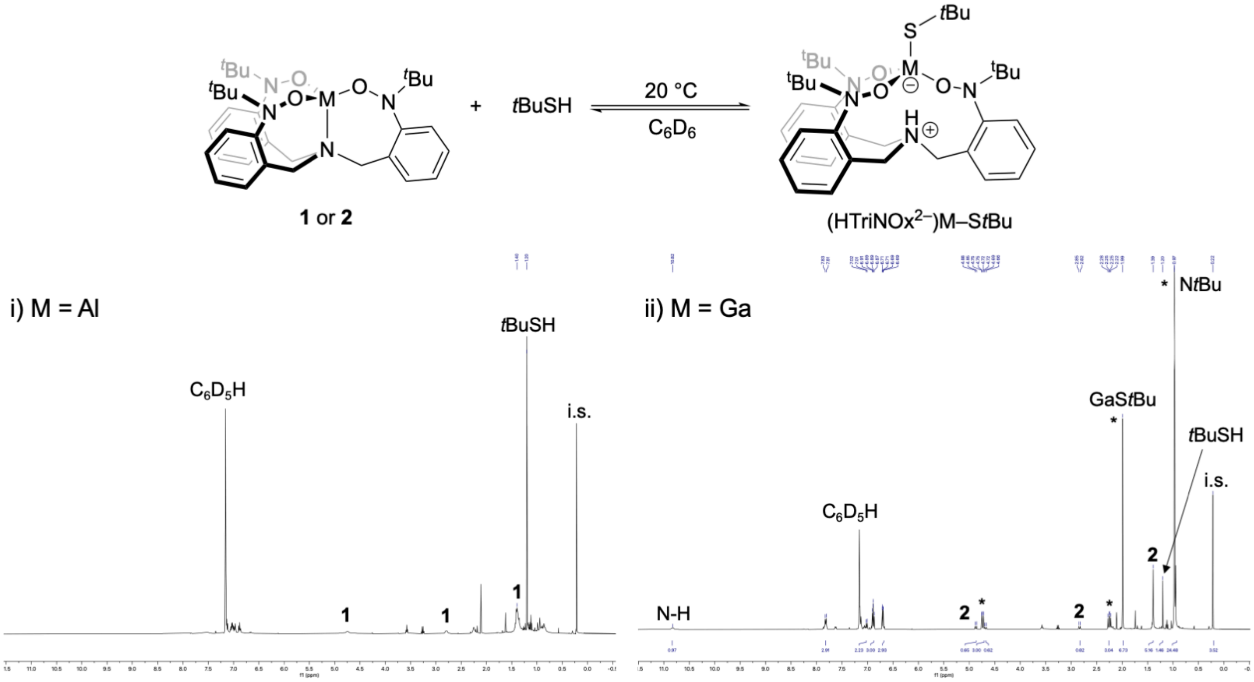

Figure S30. ^{1}H NMR spectra of the reaction of *t*-BuSH with **1** (i) and **2** (ii) in C_6D_6 recorded after 24 hours of stirring at room temperature. Resonances for the presumptive (HTriNOx^{2-})Ga–S^{t}Bu product are labeled with an *. i.s. = internal standard = hexamethylcyclotrisiloxane

Protocol for kinetic analysis of the reactions of 1 and 2 with alcohols. In the glovebox, 500 µL of a 9 mM stock solution of **1** (or **2**) in C_6D_6 was dispensed into an NMR tube along with 125 µL of a 6 mM stock solution of hexamethylcyclotrisiloxane as an internal standard, also in C_6D_6. The NMR tube was sealed with a septa-lined cap, removed from the glovebox, and transported to the NMR spectrometer which has the temperature probe set to 20 °C. Once at the instrument, 125 µL of a 36 mM stock solution of the specific alcohol in C_6D_6 was added to the NMR tube via syringe through the septa, the tube was inverted once to start the experiment (time = 0), and the NMR sample was loaded into the NMR spectrometer. Single-scan ^1H NMR spectra of the reaction were recorded at regular 2 min intervals and the concentration of the H(TriNOx^{2-})M–OR complexes were determined by integration of the protons on the apical alkoxylate ligands (-OR) against internal standard. For each experiment, $[\mathbf{1}]_0$ (or $[\mathbf{2}]_0$) = $[ROH]_0$ = 6 mM; [i.s.] = 1 mM. The total reaction volume is 0.75 mL.

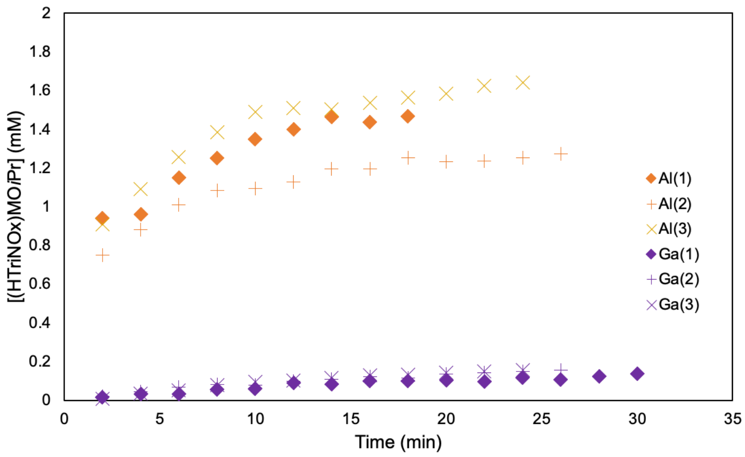

Figure S31. Plot showing the concentration of products over time for the reaction of **1** and **2** with *i*-PrOH in C_6D_6 at 20 °C.

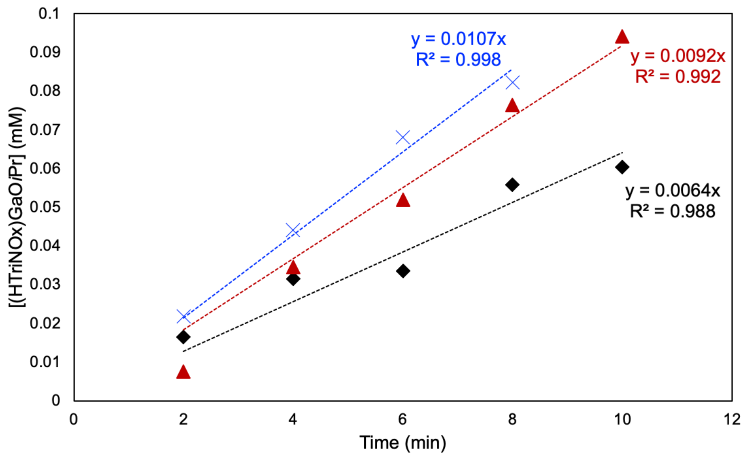

Figure S32. Initial rate data for the reaction of **2** with *i*-PrOH in C_6D_6 at 20 °C. Replicate trials are represented by blue, black, and red lines.

Procedure for the calculations to give the predicted-pK_a of alcohols in DMSO. All optimization and frequency calculations were performed with the Gaussian '16, Revision B.01 program using the G4 method[1] implementing a SCRF polarizable continuum solvent model of DMSO (ε = 46.826).

Predicted pK_a values were determined by calculating the Gibbs standard free energies for the alcohols and their corresponding alkoxide conjugate bases (Table S1). The difference of these energies for each alcohol/alkoxide pair represents the ΔG^0 of deprotonation (ΔG^0_{deprot}) for each alcohol (Table S2). The ΔG^0_{deprot} values across the range of alcohols were normalized to 2,2,2-trifluoroethanol ($\Delta G^0_{deprot,ref}$) and then converted to their corresponding calculated pK_a values via the following formula:

$$pK_a = -\ log\left[10^{\frac{-\Delta G_{deprot,ref}}{C}}\right] \qquad \text{(Eq. S3)}$$

where C is equal to [1.9872 cal/K•mol * 298.15 K * [ln(10)/1000]. These values represent the pK_a of the alcohols relative to 2,2,2-trifluoroethanol and although the absolute values hold no meaning, their relative values can be compared. To do so, we generated a calibration curve (Figure S38) between these calculated pK_a values and the pK_a values listed in the Bordwell literature for any alcohol with the latter value being available in DMSO . The line-of-best-fit equation was then used to determine the predicted pK_a values. Table S2 lists these predicted pK_a values for the range of alcohols studied along with the values from the Bordwell literature.[2]

---

[1] Curtiss, L. A.; Redfern, P. C.; Raghavachari, K. *J. Chem. Phys.* **2007**, *126*, 084108.

[2] Reich, H. Bordwell pK_a Table. https://organicchemistrydata.org/hansreich/resources/pka/

Table S1. Raw calculated Gibbs standard free energies at 298 K of alcohols and their corresponding alkoxides.

**alcohol (ROH)**	**Electronic energy + free energy correction of protonated form (hartrees)**	**Electronic energy + free energy correction of deprotonated form (hartrees)**
MeOH	-115.67961	-115.17672
EtOH	-154.96476	-154.46275
^{t}BuOH	-233.53866	-233.03685
^{i}PrOH	-194.25147	-193.75025
1-AdOH	-465.66954	-465.16899
BnOH	-346.60935	-346.11296
9-Me-FlOH	-615.64647	-615.15071
HC≡CCH_2OH	-191.79935	-191.30800
CF_3CH_2OH	-452.67243	-452.18816
CCl_3CH_2OH	-1533.42630	-1532.94569
4-MeO-C_6H_4OH	-421.80596	-421.33244
PhOH	-307.33497	-306.86398
$(CF_3)_2CHOH$	-789.65321	-789.18690

Table S2. Calculated standard Gibbs free energies of deprotonation (ΔG_{deprot}) in DMSO for alcohols and the manipulation of that data to give predicted pK_a values for alcohols.

**alcohol (ROH)**	**ΔG^0_{deprot} (kcal/mol)**	**$\Delta G^0_{deprot,ref}$[a] (kcal/mol)**	**Calculated pK_a[b]**	**Predicted pK_a[c]**	**Bordwell pK_a[d]**
MeOH	315.57	11.68	8.56	30.77	29
EtOH	315.02	11.13	8.16	30.43	29.8
^{t}BuOH	314.90	11.01	8.07	30.36	32.2
^{i}PrOH	314.52	10.63	7.79	30.12	30.25
1-AdOH	314.10	10.21	7.49	29.86	—
BnOH	311.49	7.60	5.57	28.26	—
9-Me-FlOH	311.09	7.21	5.28	28.01	—
$HC{\equiv}CCH_2OH$	308.33	4.44	3.26	26.31	—
CF_3CH_2OH	303.89	0	0	23.58	23.5
CCl_3CH_2OH	301.58	-2.30	-1.69	22.16	—
4-MeO-C_6H_4OH	297.14	-6.75	-4.95	19.42	19.1
PhOH	295.55	-8.34	-6.11	18.44	18
$(CF_3)_2CHOH$	292.61	-11.27	-8.26	16.64	17.9

a) ΔG^0_{deprot}(ROH) – ΔG^0_{deprot}(CF_3CH_2OH); b) Relative to CF_3CH_2OH.; c) Determined using the line-of-best-fit in Figure S38; d) Values are quoted in DMSO and taken from Reich, H. Bordwell pK_a Table. https://organicchemistrydata.org/hansreich/resources/pka/.

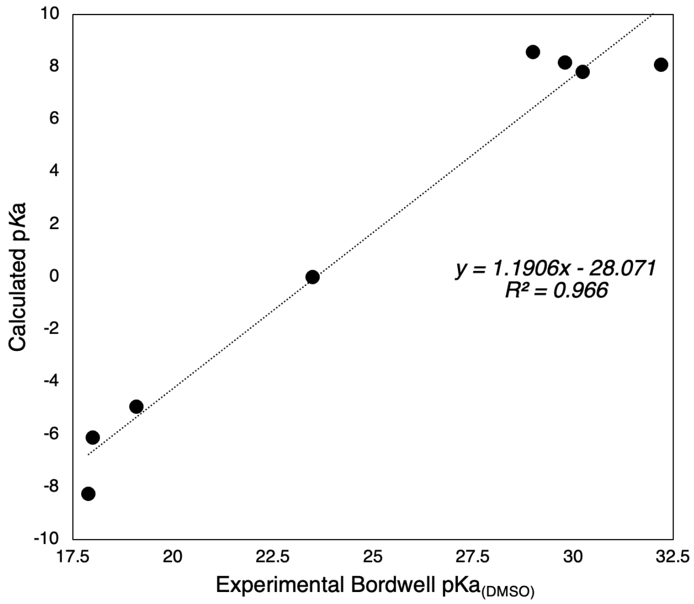

Figure S33. Correlation plot between the G4-calculated versus experimental determined pK_a values for alcohols.

Procedure for the calculations to determine the A-values for the alcohol R groups. All optimization and frequency calculations were performed with the Gaussian '16, Revision B.01 program using the G4 method.REF The geometries of both axial and equatorial conformers of the R-substituted cyclohexanes for each alcohol R group were optimized and the standard Gibbs free energy (ΔG^0, in kcal/mol) values were calculated. The A-value for a given R substituent is a measurement of how much the equatorial conformer of the R-substituted cyclohexane is favored over the axial conformer. The A-values are thus obtained by subtracting $\Delta G^0_{equatorial}$ from ΔG^0_{axial}.

Table S3. Calculated standard Gibbs free energies at 298 K of the equatorial ($\Delta G^0_{equatorial}$) and axial (ΔG^0_{axial}) conformers of R-substituted cyclohexanes and the calculated A-values for the R groups.

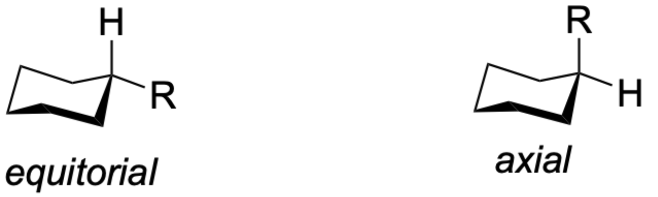

R Group	$\Delta G^0_{equatorial}$ (hartrees)	ΔG^0_{axial} (hartrees)	A-value (kcal/mol)
t-Bu	-392.880887	-392.871382	5.96
i-Pr	-353.597397	-353.593586	2.39
1-adamanyl	-625.040039	-625.035467	2.87
Me	-275.027013	-275.023177	2.41
Bn	-506.002444	-505.998587	2.42
9-MeFl	-775.083576	-775.083904	-0.21
$HCCCH_2$	-351.157602	-351.153909	2.32
CF_3CH_2	-612.065504	-612.062007	2.19
CCl_3CH_2	-1693.113611	-1693.109934	2.31
4-OMe-C_6H_4	-581.207238	-581.200354	4.32
Ph	-466.714283	-466.707497	4.26

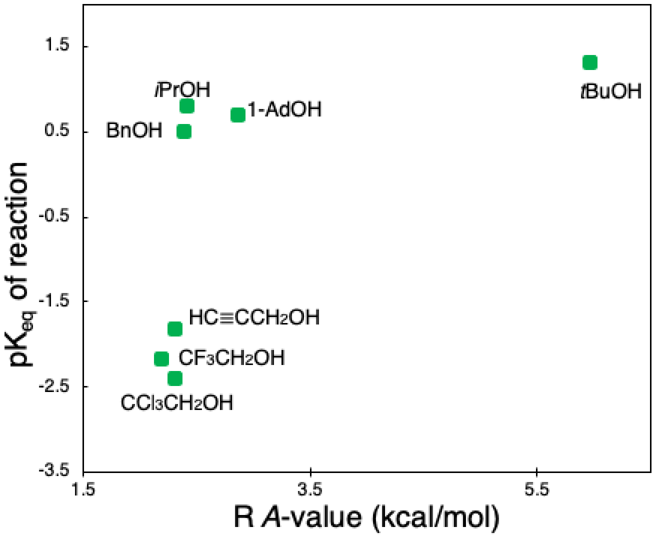

Figure S34. Plot of the pK_{eq} of the reaction of **2** with alcohol versus the A-value of the alcohol R group.
